# Proinflammatory Cytokines in Chronic Respiratory Diseases and Their Management

**DOI:** 10.3390/cells14060400

**Published:** 2025-03-09

**Authors:** Vivek P. Chavda, Rajashri Bezbaruah, Nasima Ahmed, Shahnaz Alom, Bedanta Bhattacharjee, Lakshmi Vineela Nalla, Damanbhalang Rynjah, Laura Kate Gadanec, Vasso Apostolopoulos

**Affiliations:** 1 Department of Pharmaceutics and Pharmaceutical Technology, L.M. College of Pharmacy, Ahmedabad 380009, Gujarat, India; 2 Department of Pharmaceutical Sciences, Faculty of Science and Engineering, Dibrugarh University, Dibrugarh 786004, Assam, India; rajashribezbaruah@dibru.ac.in (R.B.); ahmed.nasima91@gmail.com (N.A.); shahnazalom1@gmail.com (S.A.); 3 Institute of Pharmacy, Assam Medical College and Hospital, Dibrugarh 786002, Assam, India; 4 Girijananda Chowdhury Institute of Pharmaceutical Science-Tezpur, Sonitpur 784501, Assam, India; bedanta1994@gmail.com (B.B.); damanbhalangrynjah@gmail.com (D.R.); 5 Department of Pharmacology, GITAM School of Pharmacy, GITAM (Deemed to be University), Rushikonda, Visakhapatnam 530045, Andhra Pradesh, India; vineelavinni154@gmail.com; 6 Institute for Health and Sport, Immunology and Translational Research Group, Victoria University, Werribee, VIC 3030, Australia; laura.gadanec@live.vu.edu.au; 7 School of Health and Biomedical Sciences, RMIT University, Melbourne, VIC 3083, Australia; vasso.apostolopoulos@rmit.edu.au

**Keywords:** asthma, chemokines, chronic obstructive, COVID-19-associated lung disease, cystic fibrosis bronchiectasis, growth factors, interferons, interleukins, lung cancer, pneumonia, pulmonary fibrosis, proinflammatory cytokines, respiratory diseases

## Abstract

Pulmonary homeostasis can be agitated either by external environmental insults or endogenous factors produced during respiratory/pulmonary diseases. The lungs counter these insults by initiating mechanisms of inflammation as a localized, non-specific first-line defense response. Cytokines are small signaling glycoprotein molecules that control the immune response. They are formed by numerous categories of cell types and induce the movement, growth, differentiation, and death of cells. During respiratory diseases, multiple proinflammatory cytokines play a crucial role in orchestrating chronic inflammation and structural changes in the respiratory tract by recruiting inflammatory cells and maintaining the release of growth factors to maintain inflammation. The issue aggravates when the inflammatory response is exaggerated and/or cytokine production becomes dysregulated. In such instances, unresolving and chronic inflammatory reactions and cytokine production accelerate airway remodeling and maladaptive outcomes. Pro-inflammatory cytokines generate these deleterious consequences through interactions with receptors, which in turn initiate a signal in the cell, triggering a response. The cytokine profile and inflammatory cascade seen in different pulmonary diseases vary and have become fundamental targets for advancement in new therapeutic strategies for lung diseases. There are considerable therapeutic approaches that target cytokine-mediated inflammation in pulmonary diseases; however, blocking specific cytokines may not contribute to clinical benefit. Alternatively, broad-spectrum anti-inflammatory approaches are more likely to be clinically effective. Herein, this comprehensive review of the literature identifies various cytokines (e.g., interleukins, chemokines, and growth factors) involved in pulmonary inflammation and the pathogenesis of respiratory diseases (e.g., asthma, chronic obstructive pulmonary, lung cancer, pneumonia, and pulmonary fibrosis) and investigates targeted therapeutic treatment approaches.

## 1. Introduction

Respiratory diseases remain one of the leading causes of death in underdeveloped nations [1,2]. Alarmingly, chronic respiratory diseases are the third leading cause of death worldwide, and are responsible for 4 million deaths annually [1,2]. Despite the 2021 Global Burden of Disease Study reporting a substantial decline in mortality caused by lower respiratory infections, a considerable number of deaths in infants and young children were associated with pneumonia and other lower respiratory tract diseases [3]. Recently, the World Health Organization (WHO) has shifted focus toward implementing strategies to facilitate the prevention of noncommunicable diseases (e.g., asthma, chronic obstructive pulmonary diseases (COPD), and chronic respiratory diseases) [4], as infectious respiratory disorders can be mitigated or controlled by vaccination and medication [3]. Asthma results in approximately 455,000 fatalities each year worldwide [5], and due to its chronicity and high morbidity burden causes great disruption to an individual’s personal, social, and economic productivity, as well as quality of life [5]. Each year asthma accounts for more than 700,000 Emergency Department visits by children [6], and is believed to affect more than 350 million people globally [7]. Both developed and emerging nations have reported an increase in the prevalence of asthma [8], which may be attributed to air pollution, atmospheric chemical triggers, aeroallergens and traffic density in metropolitan areas [9,10], a lack of access to healthcare, and the availably and affordability of drugs in low-income and rural areas [11]. In addition to air pollution and workplace exposure to unhealthy air, tobacco smoke (active and passive smoke exposure) is a major contributor to the burden of respiratory diseases [12,13]. Indoor and outdoor air pollution expose almost 3 billion people to harmful consequences, resulting in 3 million avoidable deaths annually [14]. Furthermore, the frequency of lung cancer and COPD are also on the rise, with lung cancer representing the most common malignancy and the most common cause of deaths in people [15,16]. Millions of people lose their lives due to a lack of access to essential healthcare services, medications, vaccinations, and medical care [17,18]. Furthermore, as most respiratory infections are avoidable and prevention is often less expensive than the required treatment, global respiratory illness control and eradication depend on public health initiatives and surveillance, including raising awareness and increasing knowledge and education [1,19]. To find effective interventions, set targets, and effectively allocate finances and resources to minimize the burden of respiratory diseases, it is crucial to understand the socioeconomic load that these respiratory diseases place on healthcare resources [1,19]. Respiratory diseases, such as asthma, COPD, and acute respiratory distress syndrome (ARDS), are characterized by the excessive production of proinflammatory cytokines and chronic airway inflammation, which can lead to airway hyperresponsiveness, remodeling, and tissue damage [20].

**Figure 1:**
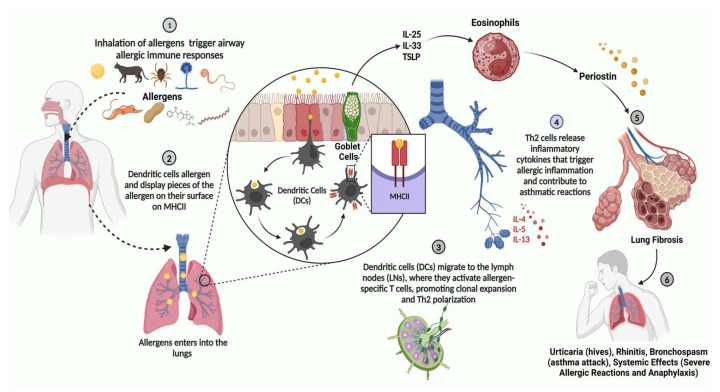
Schematic representation of the pathologic immune response in the airways. (1) Inhalation of allergens, such as pollen, dust mites, animal dander, and mold, triggers an immune reaction. (2) Dendritic cells (DCs) in the airway epithelium capture and process allergens, presenting them on MHC class II molecules [24]. (3) Activated DCs migrate to the lymph nodes, where they stimulate allergen-specific naïve T cells, leading to clonal expansion and differentiation into Th2 cells. (4) Th2 cells release inflammatory cytokines (IL-4, IL-5, IL-13), promoting eosinophilic inflammation, mucus hypersecretion, and bronchial hyper-responsiveness. (5) This cascade results in airway constriction, increased vascular permeability, and mucus production, leading to allergic asthma and, in severe cases, anaphylaxis [24]. Figure created using BioRender.com (Premium Version) (accessed on 28 February 2025).

Exposure to microorganisms, toxins, pollutants, irritants, and allergens causes airway inflammation [21]. Inflammatory receptors identify conserved molecular patterns displayed on pathogens, activating inflammatory cells such as macrophages, dendritic cells, natural killer (NK) cells, and T cells, which then release inflammatory mediators, including growth factors, chemokines, and proinflammatory cytokines [20,21,22,23]. Proinflammatory cytokines produced by immune cells in response to infection or injury, such as tumor necrosis factor (TNF) and interleukins (IL), play a significant role in the pathogenesis of respiratory diseases [20,21,22,23]. The release of cytokines triggers a cascade of events that lead to inflammation and damage to the airways and lung tissue, resulting in different respiratory illnesses and diseases [20,21,22,23]. Interestingly, while certain cytokines are proinflammatory and contribute to disease progression (e.g., IL-1, chemokines, and interferon (IFN)), others reduce inflammation and aid in the healing process (e.g., IL-4, IL-10, and IL-13), and have become of interest in cytokine biology and clinical research [24]. The mechanism of inflammation is dependent on genes (e.g., type II phospholipase A2 (PLA2), cyclooxygenase (COX)-2, and inducible nitric oxide synthase) that create small mediator molecules when inflammation occurs and that are upregulated during this process. These genes produce enzymes that boost the production of leukotrienes, prostanoids, nitric oxide (NO), and platelet-activating factor, which ultimately cause inflammation [25,26]. Endotoxins and other inflammatory products, such as histamines, reactive oxygen (ROS), and reactive nitrogen species (RNS), increase the expression of inflammatory genes and secrete IL-1 and TNF (and occasionally IFN-γ) [27] (Figure 1).

The lungs are the fundamental organs of the respiratory system, and their most basic physiological role is to facilitate gas exchange and transport [28]; however, excessive inflammation, injury, and homeostatic imbalance can be fatal [29]. Pulmonary homeostasis depends on a careful balance between proinflammatory and anti-inflammatory responses [30]. It is believed that proinflammatory cytokines secreted by inflammatory cells play a crucial role in the pathogenesis of different lung diseases, such ARDS, asthma, cystic fibrosis bronchiectasis, and COPD [22,27]. The host’s defense against pathological situations is strengthened by the proinflammatory cytokines released by macrophages [20,27]. Proinflammatory cytokines may be helpful in managing the pathology of inflammation, but when their levels increase and remain high, they can seriously harm tissue [27]. Coronavirus disease 2019 (COVID-19), caused by severe acute respiratory syndrome coronavirus-2 (SARS-CoV-2), poses a serious threat to global health [31]. Through immune system hyperactivation and uncontrolled cytokine release, COVID-19 has the ability to cause a cytokine storm in pulmonary tissues [32], similar to that observed in ARDS [33]. Therefore, managing cytokine release syndrome (CRS) and avoiding further infections may be a promising strategy for COVID-19 therapy [34]. Herein, we have attempted to establish the general role of cytokines in respiratory disorders, their signaling pathways, and their management strategies. Since most respiratory diseases, such as asthma, COPD, COVID-19-related ARDS, cystic fibrosis bronchiectasis, lung cancer, pneumonia, and pulmonary fibrosis, share similar proinflammatory cytokines, a comprehensive understanding of the inflammatory pathways related to their associated cytokines is essential to control and eradicate respiratory diseases [22].

## 2. Classification of Cytokines

Small signaling proteins generated by cells, referred to as cytokines, have a particular impact on the communication and interaction between cells [35]. These nonstructural proteins have a molecular weight that ranges from 8 to 40,000 Daltons and have an essential role in the immune system, inflammation, and healing after injury or disease [24]. Cytokines can exert their effect on the cells that release them (autocrine action), on cells that are close (paracrine action), or on cells that are far away (endocrine action) [36]. During physiological and pathological processes, immune cells, including macrophages, mast cells, T helper (Th) cells, endothelial cells, and Schwann cells, that are localized or migrate to the site of inflammation release cytokines in and via peripheral nerve tissue [37]. According to their structure, origin, and function, cytokines are classified into different types [38]. For example, (i) cytokines with chemotactic properties are called chemokines; (ii) cytokines produced by one leukocyte and acting on other leukocytes are known as ILs; (iii) cytokines with the ability to kill or inhibit tumor cells are referred to as TNF; (iv) cytokines that promote distinct stages of development and differentiation in pluripotent hematopoietic stem cells are called colony-stimulating factor (CSF); and (v) cytokines that can act by preventing lymphocyte proliferation are called transforming growth factor (TGF) [39].

### 2.1. Interleukins

Interleukins are a category of secreted proteins with a variety of forms and roles. They are predominantly produced by CD4^+^ Th lymphocytes, monocytes, macrophages, and endothelial cells [25,26]. ILs, which are effective at thymocyte and T-cell activation, proliferation, and differentiation, can also improve the killing activity of cytotoxic T (Tc) and NK cells, causing fever, stimulating hematopoiesis, and encouraging immunological responses [25]. There are currently 40 known ILs, some of which are divided into subtypes (e.g., IL-1α and IL-1β) [40]. Moreover, based on commonalities in their receptor chains and sequences and other functional characteristics, ILs can be further categorized into families. The IL-1 family consists of 11 cytokines: 3 receptor antagonists (IL-1 receptor antagonist (Ra), IL-36Ra, and IL-38); 7 agonistic ligands (IL-1α, IL-1β, IL-18, IL-33, IL-36α, IL-36β, and IL-36γ); and 1 anti-inflammatory cytokine (IL-37) [41]. Autoimmune, viral, degenerative, and autoinflammatory disorders are all closely associated with IL-1. IL-2, IL-4, IL-7, IL-9, IL-15, and IL-21 create the IL-2 cytokine family, sometimes referred to as the common γ-chain family [42]. For progenitors and mature cells, these cytokines function as growth and proliferation factors. Although dendritic cells and NK cells can also express this cytokine, CD4^+^ and CD8^+^ T cells are the major producers of cytokines [24]. As a B-cell growth factor, IL-2 stimulates the production of antibodies, aids in the generation of regulatory T (Treg) cells, and encourages the growth and differentiation of NK and Th cells. In addition to sharing some of IL-2’s properties, such as T-cell activation and NK cell proliferation stimulation, IL-15 also affects CD8^+^ memory cells and NK cell homeostasis, and increased levels of IL-15 have also been observed in autoimmune diseases [41].

### 2.2. Tumor Necrosis Factor

The term “TNF” refers to a cytokine that was first identified as a tumor necrosis-inducing molecule in 1975 and possesses the ability to kill or inhibit tumor cells [41]. TNF acts on vascular endothelial cells, causing endothelial cell damage, vascular dysfunction and injury, thrombosis, and local blood flow blockade of the tumor tissue. The TNF superfamily, which also includes the TNF receptor superfamily, has more than 40 members [43], the most notable of which are TNF-α and TNF-β (also known as lymphotoxin) [43]. TNF-α induces inflammation and causes acute phase reactions and fever. Additionally, it induces neutrophil and endothelial cell activation and kills cells via apoptotic mechanisms. The type II transmembrane protein TNF-β plays a crucial role in the formation of Peyer’s patches, in the lymph nodes, and in the maintenance of secondary lymphoid organs. Lymphocytes are primarily responsible for stimulating TNF-β expression [41].

### 2.3. Interferons

The pleiotropic characteristics of cytokines are further demonstrated by IFNs, which have the capacity to prevent viral replication [44,45,46]. Although it constitutes a broad-spectrum antiviral medication, IFN does not immediately kill or suppress an infiltrating virus. Rather, IFN induces cells to create antiviral proteins primarily through cell surface receptors, preventing hepatitis B virus from replicating [41,47]. Additionally, it can boost the health of T lymphocytes, macrophages, and NK cells, which have immunoregulatory effects, and improves the replication of antivirals [41,47]. Type I, II, and III IFNs are the three primary classes of IFNs. The two primary type I IFNs are IFN-α and IFN-β, and IFN-α is further divided into 13 distinct subtypes, including IFN-α1, IFN-α2, IFN-α4, IFN-α5, IFN-α6, IFN-α7, IFN-α8, IFN-α10, IFN-α13, IFN-α14, IFN-α16, IFN-α17, and IFN-α21 [48]. Type I IFNs have strong antiviral effects, especially against influenza, hepatitis B/C, and some RNA/DNA viruses, as well as having an important role in autoimmune diseases and cancer. IFN-α has been used therapeutically against hepatitis C, and more recently IFN-α was shown to protect against pulmonary tissue damage from the highly pathogenic avian influenza virus, H5N1 [49]. Lesser-known type I IFNs include IFN-δ, IFN-ε, IFN-ζ, IFN-κ, IFN-ω, and IFN-τ. IFN-δ is not a well-characterized IFN; however, it is expressed in response to viral infections. IFN-ε is involved in local immune responses against viruses in epithelial tissues and the mucosal surfaces of the lung and reproductive tract; IFN-ζ is expressed by dendritic cells in response to viral infections and cancer, and contributes to the stimulation of T cells; IFN-κ is expressed in keratinocytes and protects the skin against viral infections; IFN-ω is produced in response to viral infections; and IFN-τ plays an important role in pregnancy [50]. A type II IFN is IFN-γ, a proinflammatory cytokine involved in inflammation, defense against viruses, cancer, and immune regulation [50]; it also increases TNF activity and stimulates NO production [44]. Similar to type I IFNs, type III IFNs, such as IFN-λ1 (IL-29), IFN-λ2 (IL-28A), IFN-λ3 (IL-28B), and IFN-λ4, are vital in the host’s defense against viral infections, with restricted expression on mucosal tissues (respiratory, gastrointestinal, and urogenital tracts). IFN-λ also plays important roles against hepatitis, cancer, and autoimmune diseases, and has been investigated for its role as a therapeutic against viral infections.

### 2.4. Colony-Stimulating Factor

CSF is a pro-inflammatory cytokine that initiates distinct stages of development and differentiation in pluripotent hematopoietic stem cells. CSF promotes hematopoiesis, which aids in the development of monocyte, neutrophil, eosinophil, and basophil progenitors, and in the activation of macrophages [41]. Alternatively, some CSFs have the ability to stimulate or augment the phagocytosis and killing activities of neutrophils and macrophages, in addition to their role in hematopoietic stem cell proliferation and differentiation during various developmental and differentiation phases [51]. Additionally, granulocyte–macrophage CSF (GM-CSF) and macrophage CSF (M-CSF) exhibit a connection between their expression and TNF, IL-1, IL-17, and IL-23 [52].

### 2.5. Chemokines

Chemokines, which are short peptides (8000 Daltons) that facilitate the movement of leukocytes from circulation into tissues, are another group of genes that promote inflammation [53]. There are approximately 50 known human chemokines, which are divided into four groups based on the location of their N-terminal cysteine residues [54]. The two main chemokine families are C-C motif (CC) and C-X-C motif (CXC). In general, CXC chemokines are more selective for neutrophils, while CC chemokines are chemotactic for monocytes and a small proportion of lymphocytes [54]. The most well-known chemokine is IL-8, also known as CXCL8, which is a member of the CXC chemokine family and is necessary for the recruitment of neutrophils and promoting and maintaining ongoing inflammatory responses [55]. However, CCL2 (also known as monocyte chemoattractant protein-1) and CCL11 (also referred to as eotaxin) are examples of CC chemokines that affect the recruitment of a range of leukocytes, particularly monocytes and eosinophils, respectively [56]. However, involvement in inflammatory responses is the most well-known function of chemokines. Chemokines actively promote macrophage, neutrophil, and T-cell recruitment to the site of inflammation and function as secondary proinflammatory mediators brought on by primary proinflammatory mediators, such as IL-1 or TNF [41].

### 2.6. Transforming Growth Factor

TGF-β is a polypeptide growth factor that is crucial for several functions, including cell growth and differentiation, extracellular matrix synthesis, angiogenesis, apoptosis, and immune system function [57]. Monocytes and macrophages are drawn to TGF-β by its chemo-attractive properties; however, it also has an anti-inflammatory impact by preventing lymphocyte proliferation [41].

### 2.7. Other Cytokines

In addition to the previously described cytokines, some other types of cytokines that play important roles in the pulmonary inflammatory process include leukemia inhibitory factor and oncostatin M, which cause the production of acute-phase proteins [58,59]. Moreover, osteopontin promotes the development of IL-2 and inhibits the production of IL-10 [41]. Table 1 summarizes the classifications of cytokines, along with their origins of secretion and roles during inflammation.

## 3. Pro-Inflammatory Cytokines in Respiratory Diseases

The lungs and airways are constantly exposed to external airborne insults comprising a mixture of harmless inhaled particles and detrimental pathogens, aeroallergens, and pollutants [20]. Cytokines play a pivotal role in the pulmonary equilibrium and have the ability to regulate host responses to damage or infections within the lungs, leading to the removal of injury, repair of lung tissue, and restoration of homeostasis [20]. However, as cytokine-mediated tissue damage can compromise gas exchange and transport, it is essential that pulmonary immunity is tightly controlled, enabling pathogenic clearance and the prompt restoration of homeostasis, while simultaneously avoiding unnecessary excessive and chronic inflammatory responses [20]. When the inflammatory response is unregulated or cytokine production becomes unbalanced, deleterious consequences occur, including airway remodeling, fibrosis, and undesired, maladaptive effects [63]. Proinflammatory cytokines secreted by inflammatory cells play a crucial role in the pathogenesis of different lung diseases, such as ARDS, asthma, cystic fibrosis bronchiectasis, and COPD (Table 2) [22]. Cytokines generated by Th2 cells appear crucial in directing inflammation, at least in mild-to-moderate illness [64]. Although there are many respiratory conditions that arise due to underlying inflammatory cytokine release, we have highlighted several major respiratory conditions, including COPD, asthma, pulmonary fibrosis, cystic fibrosis bronchiectasis, COVID-19, pneumonia, and lung cancer, that align with the major acting cytokines (Table 2).

In the context of chronic respiratory disease (asthma, COPD, etc.) extended exposition to inflammatory signals might eventually cause T-cell depletion and a decrease in responsiveness. High amounts of inhibitory receptors like PD-1, TIM-3, LAG-3, and TIGIT are frequently expressed by these cells’ exhaustion. This also reduces the generation of cytokines such as IFN-γ, TNF-α, and IL-2, which are essential for efficient immune system responses. However, they may also cause low-to-no response to therapeutic treatments, for instance immunotherapy, which later depend on a vigorous immune response [64].

### 3.1. Chronic Obstructive Pulmonary Disease

**Figure 2:**
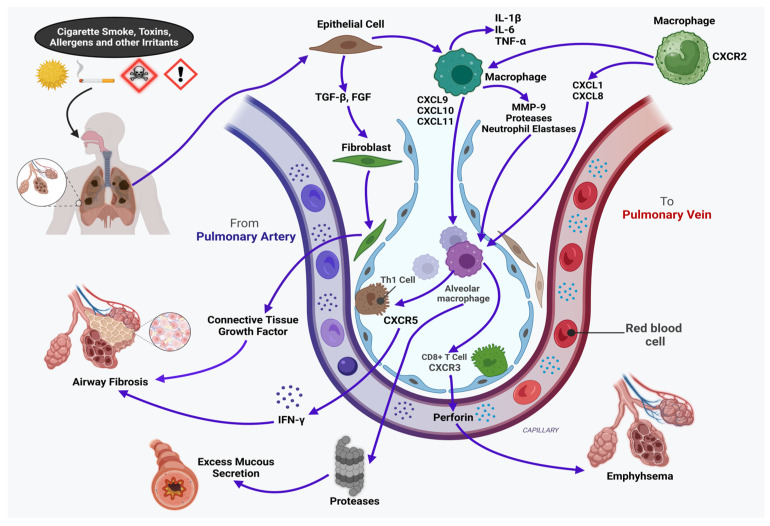
The role of cytokines in COPD. (1) Inhaled irritants, such as cigarette smoke, cause macrophages and epithelial cells to release several cytokines, including growth factors such as TGF-β and FGFs. These factors encourage fibroblast proliferation, which leads to fibrosis in the small airways. (2) These cells also release a number of chemokines that draw circulating cells into the lungs, as well as the proinflammatory cytokines TNF-α, IL-1β, and IL-6, all of which increase inflammation. (3) Monocytes are drawn to CCL2 via CCR2. (4) Neutrophils and monocytes are drawn to CXCL1 and CXCL8 via CXCR2. (5) Th1 cells and Tc1 cells are drawn to CXCL9, CXCL10, and CXCL11 via CXCR3; both of these cell types release IFN-γ, which in turn promotes the production of further CXCR3-binding chemokines [87]. (6) Macrophages and neutrophils secrete neutrophil elastase MMP-9 to disrupt the pathogens and tissues of the host, which leads to emphysema and an excess secretion of mucus. Abbreviations: COPD, chronic obstructive pulmonary disease; CTGF, connective tissue growth factor; FGF, fibroblast growth factor; IFN, interferon; IL, interleukin; Tc, cytotoxic T cell; TGF; transforming growth factor; Th, T helper; TNF, tumor necrosis factor. Figure created using BioRender.com (Premium Version) (accessed on 21 February 2025).

Smoking and extended exposure to fumes, dust, or polluted air are the main causes of COPD, which manifests as chronic bronchial inflammation, lung tissue damage (emphysema), and some degree of lung fibrosis (Figure 2). The traditional proinflammatory cytokines (i.e., TNF-α, IFN-γ, IL-1, IL-6, IL-8, IL-18, and IL-32) drive the inflammatory process involving T cells, neutrophils, and macrophages; among these, IL-8 provides an integral function in the inflammatory reaction and stimulates neutrophils to the spot of inflammation in the lungs. Additionally, recent research has highlighted IL-17 in the pathophysiology of COPD [63]. Patients with COPD have elevated sputum IL-17 levels that are significantly more pronounced than those in patients with asthma [68]. Activated Th1 and Th2 cells were observed in COPD patients, suggesting that both may contribute to the disease processes, even though COPD is not often thought of as a Th2-mediated disease. Similarly, IL-13 may cause vascular remodeling, airway fibrosis, and mucus metaplasia in COPD [69]. Patients with COPD have an increased expression of the profibrotic cytokine TGF-β in their airways, and TGF-β is believed to help alter the structural composition of the lungs [63,65]. A new cytokine target in COPD that has gained notoriety is the D-dopachrome tautomerase (D-DT)/atypical chemokine-receptor-3 (ACKR3) ligand/receptor axis. Prior research has shown that D-DT and macrophage migration inhibitory factor (MIF) have conflicting roles in COPD. MIF also appears to have tissue-protective roles in myocardial ischemia, metabolic liver disease, and alveolar regeneration, although it is often believed to have proinflammatory, disease-exacerbating effects. Although D-DT has protective benefits against the inflammation of adipose tissue, heart failure, and myocardial ischemia, it also encourages endotoxemia, multiple sclerosis, and carcinogenesis. ACKR3, a new receptor for D-DT, was implicated by Melgert’s group as having a protective role for D-DT in COPD. The findings have implications for utilizing pro-repair D-DT as a COPD treatment plan [70,71]. During clinical trials, IL-blocking antibodies have been reported to reduce the number of acute exacerbations and methods that inhibit upstream cytokines, such as thymic stromal lymphopoietin (TSLP), and have the potential to improve patient health in COPD [65].

### 3.2. Asthma

**Figure 3:**
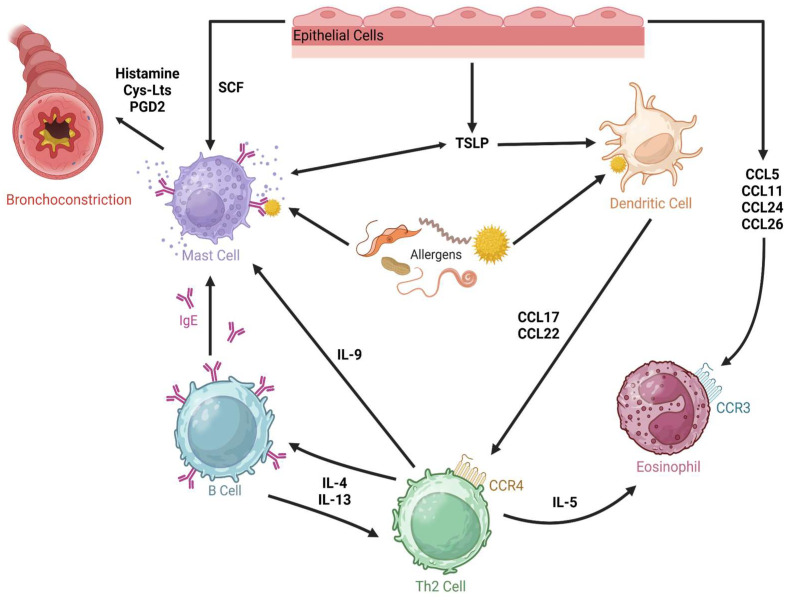
The role of cytokines in asthma. Due to the release of numerous cytokines, such as SCF (which keeps mast cells in the airways), TSLP (which acts on dendritic cells to release the Th2 chemoattractants CCL17 and CCL22 to act via CCR4), and several chemokines that attract eosinophils by activating CCR3, epithelial cells play a significant role in effectively managing inflammation in asthma. Th2 cells control the inflammatory response in asthma by releasing IL-4, IL-13, IL-5, and IL-9, which encourage eosinophilic inflammation and drive B cells to produce IgE, resulting in the stimulation of mast cell proliferation. By releasing histamine, Cys-LTs, and PGD2, mast cells are controlled by a number of interrelated cytokines and play a significant role in asthma [64]. Abbreviations: Cys-LTs, cysteinyl-leukotrienes; Ig, immunoglobulin; IL, interleukin; PGD_2,_ prostaglandin D 2; SCF, stem cell factor; Th, T helper; TSLP, thymic stromal lymphopoietin. Figure created using BioRender.com (Premium Version) (accessed on 17 November 2024).

Asthma is a persistent inflammatory condition of the airways that is accompanied by structural alterations, such as an increase in smooth muscle and the remodeling of the airway wall [64]. Asthma is defined by significant fluctuations in airway function that are caused by significant interactions with the environment, such as exposure to allergens, pollutants, and viruses, in addition to underlying chronic mediator release (Figure 3) [64]. In mild-to-moderate allergic asthma, type 2 CD4^+^ lymphocytes (Th2 cells) and their cytokines predominate, whereas severe steroid-resistant asthma has a more mixed Th2/Th1 phenotype with a Th17 component. Additionally, compared to eosinophilic and pauci-granulocytic asthma, neutrophilic asthma has been shown to have higher levels of IL-1β [66]. Other immune cells, such as neutrophils, macrophages, and dendritic cells, as well as structural cells, including the epithelium and smooth muscle cells in the airways, also release cytokines that lead to the initiation and progression of asthma. Asthma is distinct from other chronic inflammatory conditions, such as psoriasis, Crohn’s disease, and rheumatoid arthritis, in that it displays a distinctive cytokine response characterized by Th2 cytokines, the bulk of which are encoded in a small cluster on chromosome 5q32-34. It has been proposed that the coordinated control of the immune response in favor of Th2 cytokines, such as IL-3, IL-4, IL-5, IL-6, IL-9, IL-13, and GM-CSF, is caused by a decrease in the inhibitory influence of Th1 cytokines, particularly IL-18, IL-12, and IFN. This imbalance between Th1- and Th2-type immunity appears early in childhood and may even start before birth in people who are predisposed to developing atopic dermatitis [67]. Clinical studies have shown elevated numbers of these immune cells and cytokines, and research employing asthma-prone mouse models has suggested their probable involvement in illness. In clinical trials using cytokine inhibitors, there have been reports of success in the treatment of inflammatory respiratory diseases. Combination medicines that inhibit several cytokines (such as corticosteroids) and a variety of redundant and disjointed pathways that each individually contribute to the pathophysiology of asthma may be the most efficient treatments for inflammatory diseases such as asthma, in contrast to acute diseases where inflammation is a transient reaction. Asthma entails persistent inflammation that, gradually, may result in immune cell exhaustion. This can lead to reduced therapeutic responses and prolonged appearances [64,65,88].

### 3.3. Pulmonary Fibrosis

The evolution of a number of lung disorders can ultimately lead to pulmonary fibrosis, defined as an excessive accumulation of scar tissue [89]. In contrast to acute respiratory situations, where inflammation is a transient reaction, pulmonary fibrosis is characterized by persistent inflammation that causes lung tissue to eventually scar (fibrosis). The inflammation in pulmonary fibrosis is characterized by the activation of fibroblasts through several pathways, like the TGF-β/Smad pathway, epithelial–mesenchymal transition, and oxidative stress, which causes tissue stiffening and scarring. The fibrotic process may be self-limiting in some instances; however, it can also become overt and irreversible, causing severe loss in lung function and gas exchange capacity [89]. While idiopathic pulmonary fibrosis patients have a median survival of just two to three years, pulmonary fibrosis is the primary cause of death in patients with the autoimmune disease scleroderma [90]. Collagen and extracellular matrix build up in the lungs in a variety of ways in pulmonary fibrosis [89]. Lung fibrosis is also influenced by the process known as epithelial–mesenchymal transition, which transforms lung epithelial cells into myofibroblasts [89]. All of these mechanisms are regulated by cytokines, either by directly attracting and activating cells that produce extracellular matrix or indirectly by regulating pulmonary inflammation, redox balance, and the activity of various enzymatic systems, such as matrix metalloproteinases (MMP) and their inhibiting and clotting enzymes [63].

Unquestionably, the most potent profibrotic cytokine is TGF-β. It is created in inactive form and typically kept in this state in tissues and the extracellular matrix; however, to exercise its functional impact, it must be activated [29]. The mechanism of fibrosis in the lungs is largely dependent on the activation of TGF-β by αV-containing integrins [63,85]. Together with TGF-β, connective tissue growth factor promotes fibrosis. Additionally, the Th2 cytokines IL-4 and IL-13 are powerful direct inhibitors of extracellular matrix deposition, whereas the Th1 cytokine IFN-γ is a direct promoter of fibrosis [63,72]. In contrast to scleroderma lung disease and idiopathic pulmonary fibrosis, patients suffer from a “profibrotic cytokine storm” with high levels of all cytokines. Th2 cytokines are the main drivers of fibrosis in asthma. In addition, TGF-β is a more potent profibrotic cytokine than IL-4 or IL-13, acting at considerably lower quantities. Utilizing both direct and indirect processes, the chemokine monocyte chemotactic protein (MCP)-1/CCL2, oncostatin M, and platelet-derived growth factor (PDGF) induce fibrosis. Pulmonary and activation-regulated chemokine (PARC)/CCL18 is a significant, primarily indirect, modulator of fibrosis and is elevated in association with various fibrotic lung diseases, including scleroderma, hypersensitivity pneumonitis, idiopathic pulmonary fibrosis, asthma, and sarcoidosis. Stromal cell-derived factor-1 and CXCL12 are other chemokines that cause fibrosis by attracting bone-marrow-derived fibroblast progenitors to the lung. The precise involvement of several other cytokines, including IL-1b, IL-17, IL-10, thymus and activation-regulated chemokine (CCL17), and GM-CSF, in the process of pulmonary fibrosis is still debatable or mechanistically unknown [63].

### 3.4. Cystic Fibrosis Bronchiectasis

Cystic fibrosis (CF) bronchiectasis is normally characterized by persistent and intense inflammation as a result of dysregulation in the inflammatory mediators, which begins early in life and develops throughout a patient’s life [74,76]. The inflammation is also predominantly due to neutrophil stimulation and oxidative stress, which later exacerbate inflammation and tissue damage by activating the NF-κB pathway, leading to chronic inflammatory response. Additionally, the mitogen-activated protein kinase (MAPK) signaling pathways and the CFTR gene mutation contribute to the inflammatory response, oxidative stress, and inflammation on the affected site [76]. IL-1β is a proinflammatory mediator that is upregulated in respiratory conditions, such as cystic fibrosis bronchiectasis, COPD, and asthma [74]. In exposure to cigarette smoking, bronchial epithelial cells produce IL-1β, causing goblet cell hypertrophy, fibrosis, emphysema, and pulmonary inflammation in rodents [76]. IL-1β concentrations in bronchoalveolar lavage (BAL) fluid may be elevated in response to exposure to *Pseudomonas aeruginosa*, one of the primary therapeutically prominent microbes in cystic fibrosis bronchiectasis [76,91]. Furthermore, it has been shown that the intensity of the condition is correlated with mutations in IL-1β genes [78]. In contrast to these observations, Muselet-Charlier et al. reported a rapid IL-1β-induced stimulation of nuclear factor kappa-light-chain-enhancer of activated B cells (NF-κB) in a cystic fibrosis bronchial epithelial cell line [77]. Therefore, neutralizing or inhibiting IL-1β cytokines may be a successful treatment for different inflammatory respiratory diseases [77].

### 3.5. COVID-19-Related Respiratory Disease

SARS-CoV-2, which is responsible for causing the COVID-19 pandemic, has created devastating social and economic disruption and placed immense pressure on national and global healthcare systems [92]. Due to its ability to affect the respiratory tract, the SARS-CoV-2 virus typically causes pneumonia in the majority of patients and ARDS in 15% of cases [93]. One of the major causes of death in COVID-19 patients is ARDS, which has been attributed to a robust cytokine storm clinically defined as episodes of increased proinflammatory cytokine levels [34,94]. Acute lung injuries, cellular damage, microbial modification, severe lymphopenia, and a decrease in regulatory T cells are just a few of the serious effects of excessive cytokine production [95]. In patients with ARDS, the respiratory epithelium becomes compromised due to an increased release of pro-inflammatory cytokines in the lungs, such as IL-1β, IL-6, IL-17, and TNF-α, which play a critical role in lung injury [73].

Several proinflammatory cytokines, including IL-1β, IL-6, IL-8, IL-17, and TNF-α, contribute to the pathogenesis of CRS [96]. After infection with SARS-CoV-2, numerous cells, including epithelial cells, macrophages, T lymphocytes, neutrophils, and Th17 cells, release these proinflammatory cytokines in an unregulated manner, which worsens SARS-CoV-2 infection [97]. The main receptor utilized by SARS-CoV-2 is angiotensin-converting enzyme-2, which initiates various signaling cascades that encourage the production of inflammatory cytokines [98,99]. Viral proteins have been shown to trigger the release of IL-6, which activates the transcription factor NF-κB [100]. Pro-inflammatory cytokines are released when the IL-6/soluble IL-6 receptor (sIL-6R) complex interacts with the gp130 protein, initiating a variety of intracellular signaling pathways, including Janus kinase (JAK)/signal transducer, transcription activation (STAT), and phosphatidylinositol 3-kinase (PI3K) [101]. This causes the release and transport of p65 and p50 proteins to the nucleus as a result of the phosphorylation of NF-κB inhibitor proteins, which then triggers NF-κB transcription and TNFα production [101]. Viral proteins, such as open reading frame 3a and protein E, activate the NLR-domain-containing protein 3 inflammasome, promoting the release of IL-1β [102]. CD4^+^ T cells can differentiate into Th17 cells and produce IL-17 because of their association with IL-1β, IL-23, IL-6, and TGB-β. This differentiation may also be favored by hypoxia [103]. Activation of the C-Jun N-terminal kinase (JNK), p38, or extracellular signal-regulated kinase (ERK) initiates the translocation and activation of activator protein 1 (AP-1) on the IL-8 promoter, which results in the synthesis of IL-8. Neutrophils are drawn to the infectious site by the actions of the cytokines IL-8 and IL-17, which further causes inflammation and tissue damage [97]. Since there is no specific treatment for COVID-19 in critically ill patients, COVID-19 has yet to be completely eradicated; however, reducing the inflammatory response may be a viable option [73].

### 3.6. Pneumonia

In addition to other respiratory diseases, inflammatory cytokines are also responsible for the pathogenesis of pneumonia and lung cancer. Despite the availability of antibacterial medicines, diagnostic techniques, and sophisticated instruments, bacterial pneumonia remains a significant source of global morbidity and mortality [104]. The imbalance between proinflammatory and anti-inflammatory cytokines leads to the progression of pneumonia [83]. Macrophages are the first line of defense against pathogens, performing phagocytic activity, releasing a variety of pro- and anti-inflammatory cytokines, and shaping the tissue microenvironment [83]. Throughout the course of pneumonia, alveolar macrophages and infiltrating blood monocytes release increasing levels of cytokines, which not only activate antiviral or antibacterial immunity, but also raise the danger of a cytokine storm and tissue damage [83]. Pathogen-associated molecular patterns, such as lipopolysaccharide (LPS), bacterial or viral DNA, and some cytokines (i.e., IFN), induce polarization toward pro-inflammatory (M1-like) macrophages via STAT1, NF-κB, and IFN regulatory factor transcription factor signaling, resulting in augmented pro-inflammatory cytokine production (e.g., IL6, IL1β, IL8, IL33, TNF-α, and IFN-1) [105,106,107]. The attraction of other immune cell subsets, acute inflammation, antipathogen defense, and cytokine storms are all functions of M1-like monocytic cells. The cytokines IL4, IL13, and TGF induce the anti-inflammatory (M2-like) polarization of macrophages, which leads to inflammation resolution, tissue remodeling, and regeneration [107]. For optimal pathogen clearance and efficient structural and functional healing, the balance between M1/M2 states is necessary [108].

According to studies investigating community-acquired pneumonia, blood concentrations of TNF-α, IL-lβ, IL-8, IFN-γ, and IL-6 were higher in infected individuals when compared to healthy volunteers, which indicates that they play a significant role in inflammatory responses [109,110]. While serum IL-6 levels were highest in patients with *pneumococcal pneumonia* and pneumonia caused by a *Legionella* species, serum TNF levels were higher in the majority of patients, regardless of the etiology [82]. None of the patients with *Chlamydia pneumoniae*, *Moraxella catarrhalis,* or *Haemophilus influenzae*-related pneumonia had serum IL-6 levels greater than 500 pg/mL [82]. The greatest levels of serum IFN-γ were observed in patients with pneumonia caused by Legionella species and Chlamydia pneumoniae. In contrast, serum IFN-γ concentrations were elevated in the majority of patients with viral or intracellular bacterial illness [82]. Moreover, Kragsbjerg et al. found that patients with bacteremic *Pneumococcal pneumonia* had significantly greater blood IL-8 levels on admission than patients with *Chlamydia pneumonia*, *Legionella pneumonia*, or influenza A virus infection [111]. Severe sepsis is another complication associated with community-acquired pneumonia. It has been found that the levels of IL-6 and TNF (proinflammatory cytokines) are higher in severe sepsis than those of IL-10 (anti-inflammatory cytokines) [81]. In non-acquired immunodeficiency syndrome immunocompromised patients with *Pneumocystis jirovecii* pneumonia (PcP), there was an imbalance between pro-inflammatory and anti-inflammatory cytokines in the BALF and blood [84]. In comparison to normal individuals, PcP patients exhibited significantly greater BALF levels of IL-1, TNF-α, IL-6, IL-8, and MCP-1, as well as significantly higher blood levels of IL-10, TGF-1 β, IL-8, IL-6, and MCP-1 [84].

### 3.7. Lung Cancer

Inflammation is a necessary reaction that is initiated in order to heal tissue injury and neutralize and eliminate pathogenic invaders; however, uncontrolled inflammation can become chronic, causing malignant cell changes in the surrounding tissue [112]. Inflammation increases the bioactive molecules in the tumor microenvironment, such as cytokines, chemokines, growth factors, and matrix-modifying enzymes (e.g., MMPs), which in turn raises the risk of cancer development [80]. Lung cancer remains one of the most prevalent types of cancer and has a high fatality rate [15,16]. Smoking and hereditary factors play a critical role in the development of lung cancer. Furthermore, the tumor microenvironment is also important in the growth and progression of lung cancer [113]. In lung cancer, cytokines are crucial in stimulating the body’s immune system, and can either promote tumor growth (oncogenic cytokines) or inhibit tumor growth (antitumor cytokines) by influencing the signaling pathways involved in proliferation, growth, metastasis, and apoptosis [79]. There are several routes that drive lung cancer, including the NF-κB pathway, which produces pro-inflammatory cytokines that aid in the endurance and spread of tumor cells. Histone alterations and DNA methylation can control inflammatory pathways in lung cancer, whereas epigenetic modification and IL-6/STAT3 enhance tumor development, metastasis, and angiogenesis. These changes may cause inflammatory mediators to be overexpressed and promote the development of tumors. In lung cancer patients with carcinomatous pleurisy, the levels of IL-6 and IL-8 are higher, and the soluble form of sIL-6R is lower in effusion fluid compared to normal individuals [114]. TNF-α, IFN-γ, TGF-β, vascular endothelial growth factor, and ILs (e.g., IL-6, IL-17, IL-8, IL-10, IL-22, IL-1β, and IL-18) are the most relevant cytokines involved in lung cancer [80,115,116,117].

### 3.8. Tuberculosis

Tuberculosis (TB) is an airborne ailment triggered by the bacteria *Mycobacterium tuberculosis* (Mtb) and spread via tiny respiratory aerosols from an infected individual. The bacteria Mtb typically reaches the pulmonary alveolus via the aerosol delivery of particles ranging from 2 to 5 m. Mtb interacts with neutrophils, dendritic cells (DCs), alveolar macrophages, alveolar type II pneumocytes, and airway epithelial cells (AECs) in this microenvironment [118]. Both alveolar type II pneumocytes and AECs are expected to aid in the initial host immune defense after coming into interaction with Mtb by generating cytokines, chemokines, and other chemicals that may either promptly kill Mtb or improve the antimicrobial capabilities of infected macrophages. Furthermore, when infected DCs migrate to nearby lymph nodes, an innate immune response is triggered, which is crucial to halt the bacterium’s development and convert it into a dormant form [119].

Multifunctional cytokines coordinate all of these responses, and some cytokines have been shown to have pro- or anti-inflammatory properties that lead to immune-mediated illness and infection. The cytokines that have been reported to produce Mtb infection are TNF-α, IL-1β, IL-18, IL-6, IL-17, and IL-22. The main producers of TNF-α in the case of Mtb infection are AECs, alveolar type II pneumocytes, NK T cells, macrophages, alveolar macrophages, DCs, neutrophils, CD4^+^ (Th1), and CD8^+^ T cells; similarly, IL-17 and IL-22 are produced by CD4^+^ (Th17) T cells and NK T cells, which recruit and activate phagocytic cells and cause proinflammation in the lungs of infected individuals. Macrophages and DCs can produce IL-1β, IL-18, and IL-6, which stimulate the initiation of NK T cells. This results in IFN-γ secretion from Th1 cells and is followed by proinflammation at the infected site. However, several other cytokines are also produced, like TNF- β, IL-10, and IFN-γ, which are responsible for anti-inflammatory effects [86].

It is well recognized that immunosuppressive cytokines, such as IL-10, influence immune cells and encourage Mtb infection. Studies conducted on macrophages revealed that IL-10 levels decreased when Histone Deacetylase 6 (HDAC6) expression decreased and HDAC11 expression spiked, indicating that the former is a transcription activator while the latter type is an IL-10 suppressor [120].

During Mtb H37Rv infection, C-C chemokine receptor type 5 and extracellular signal-regulated kinase (CCR5/ERK)-regulated histone phosphorylation as well as acetylation reduce the development of MHC-II in macrophages. Without uncertainty, the absolute most recognized human immune factor that fights mycobacteria is TNF-α. As a result, Mtb might defeat a host’s fight against TB by expressing certain mycobacterial proteins and lowering TNF-α production [121].

## 4. Characteristics of Cytokine Receptor Interactions

**Figure 4:**
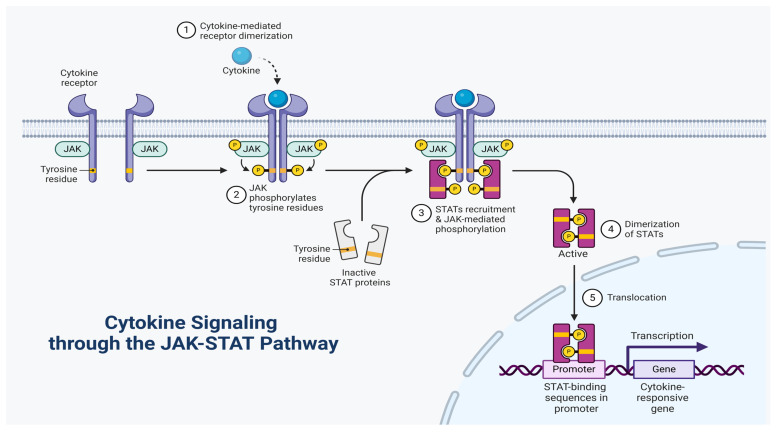
Schematic representation of cytokine signaling through the JAK/STAT cascade with a focus on cytokine receptor interactions. The process begins with cytokine-mediated receptor dimerization, where a cytokine binds to its receptor, leading to the dimerization of receptor subunits, a crucial step in intracellular signaling activation. Following this, JAK phosphorylates tyrosine residues on the intracellular domain of the receptor, creating docking sites for signaling proteins. Next, STAT proteins are recruited to the phosphorylated receptor and undergo JAK-mediated phosphorylation, enabling their activation. Once phosphorylated, STATs dimerize, a necessary step for their full activation. The active STAT dimers then translocate into the nucleus, where they bind to specific promoter sequences of cytokine-responsive genes, initiating transcription and driving various cellular responses. This pathway plays a fundamental role in immune regulation, inflammation, and cell growth. Figure created using BioRender.com (Premium Version) (accessed on 21 February 2025).

Numerous immunoregulatory effects of cytokines are vital to human biology and disease. However, the anticipated immunotherapeutic benefits of native cytokines are frequently diminished by toxicity or a lack of efficacy, either of which may occur from cytokine receptor pleiotropy and/or the unintentional activation of off-target cells [122]. Numerous crucial aspects of immunological function and many other facets of mammalian physiology are regulated by helical cytokines. The cytokine–receptor complex initiates the activation of intracellularly associated Janus kinases (JAKs) in the classical cytokine signaling pathway [123]. STAT factors are then phosphorylated and activated by JAKs to modify gene expression and ultimately influence cell fate. Some cytokines can activate the protein kinase B (Akt) and ERK pathways in addition to JAK/STAT signaling (Figure 4) [124,125]. Ligand-mediated dimerization is the standard method of cytokine receptor signaling. Regardless of cytokine receptors existing as dimers in their unliganded state, the signaling complex still requires two JAK-associated receptor subunits to bind to the cytokine at the same time to initiate signaling [122]. Tyrosine kinase (TK) receptors also exhibit a dimerization paradigm, and it appears that virtually all single-pass transmembrane receptors require ligand-mediated oligomerization or rearrangement to begin signaling [122]. The majority of cytokines form heterodimeric receptor complexes with a shared receptor subunit, such as the common gamma chain, gp130, and common beta chain (βc), together with a ligand-specific component (e.g., IL-2Rβ, IL-6Rα, or IL-3Rα) [126,127].

**Figure 5:**
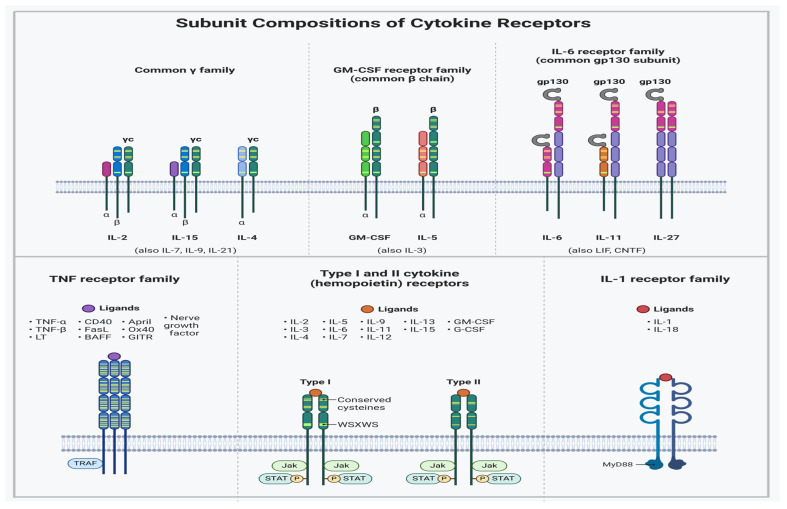
Fundamental structural elements of cytokine receptors. The figure provides a comprehensive overview of cytokine receptor subunit compositions, categorizing them into major receptor families based on their structural similarities and signaling mechanisms. The Common γ (gamma) Family includes receptors for IL-2, IL-15, and IL-4, which also interact with IL-7, IL-9, and IL-21. These receptors share a common γ-chain (γc) as a crucial signaling subunit, along with additional α and β subunits. The GM-CSF Receptor Family, also known as the Common β Chain family, consists of receptors for GM-CSF, IL-5, and IL-3, all of which rely on a shared β-chain for signal transduction. The IL-6 Receptor Family, characterized by the common gp130 subunit, includes receptors for IL-6, IL-11, IL-27, as well as LIF and CNTF, which play significant roles in cytokine-mediated signaling. The TNF Receptor Family binds ligands such as TNF-α, TNF-β, FasL, BAFF, and nerve growth factor (NGF), and its signaling pathways often involve TRAF (TNF receptor-associated factor), which mediates inflammatory and apoptotic responses. Type I and II cytokine (Hemopoietin) receptors include type I receptors, which recognize cytokines such as IL-2, IL-3, IL-4, IL-5, IL-6, IL-7, IL-9, IL-11, IL-12, IL-13, IL-15, GM-CSF, and G-CSF, and type II receptors, which signal via the JAK-STAT pathway. Type I receptors are structurally defined by conserved cysteine residues and WSXWS motifs that are essential for function. Lastly, the IL-1 Receptor Family, which includes receptors for IL-1 and IL-18, signals through the MyD88 adaptor protein, contributing to innate immunity and inflammatory responses. Overall, these receptor families share common subunits that facilitate ligand binding and signaling, with JAK-STAT signaling being predominant in type I and II receptors, TNF receptors playing key roles in inflammation and apoptosis, and IL-1 receptors utilizing MyD88-dependent pathways for immune activation. Figure created using BioRender.com (Premium Version) (accessed on 21 February 2025).

Cytokines interact with loops originating from conjoined fibronectin type III domains that make up the cytokine-binding homology regions (CHRs) of receptor chains by using the sides of their helical faces. The site 1/site 2 binding architecture, in which the cytokine binds to a first receptor subunit through site 1 and then recruits a second receptor subunit through site 2, was initially established in early structural studies on the human growth hormone system. This fundamental structural component can be seen in some form in every cytokine–receptor complex structure [128,129]. Erythropoietin and type I IFNs are two cytokines that, in contrast, activate each receptor chain in more energetically independent ways, as receptor–receptor stem interaction is minimal in these cytokine complexes [122]. There are numerous variants of the site 1/site 2 paradigm, as shown by the present database of crystal structures for cytokine–receptor complexes [130]. Site 3 is the third binding site identified in cytokines of the gp130/IL-6 class and is located at the tip of the α-helical bundle. It interacts with an immunoglobulin (Ig)-like domain on top of CHRs of the tall receptor class, for example, gp130, leukemic inhibitory factor (LIF) receptor, granulocyte colony-stimulating factor receptor (GCSF-R), IL-12Rβ2, and IL-23Rα. An interwoven c receptor dimer in the c family produces a site 2 interface that is a hybrid binding site created by the union of two head-to-head antiparallel βc chains. Gp130- and βc-class cytokines are able to build signaling complexes that are of a higher order than the typical 1:2 cytokine–receptor stoichiometry due to these structural differences. The fundamental cytokine–CHR recognition unit, for instance, is doubled by site 3 contact in the tetrameric GCSF/GCSF-R, viral IL-6/gp130, and hexameric IL-6/IL-6R/gp130 signaling complexes. Research targeting site 3, aimed at developing inhibitors of gp130 family cytokines such as IL-6, IL-23, and LIF, has been widespread. Instances where granulocyte–macrophage colony-stimulating factor (GM-CSF) is coupled to the GM-CSF receptor and βc appear to be driven by an extensive network of other interactions in the βc system. These interactions could be explicitly targeted when developing βc cytokines. A third Ig domain of their alpha receptors, which is positioned on top of the helical bundle and is present in a number of cytokines (e.g., IL-5 and IL-13), clearly represents an engineerable location [122,131,132]. IL-2 and IL-15 are the only ILs known to have a third receptor component (such as IL-2Rα and IL-15Rα, respectively) that is distinct from normal CHR-containing receptors in terms of both structure and cytokine interaction. These extra alpha subunits appear to be largely used to increase affinities and modify the target cell selectivity of their respective cytokines because they lack well-defined signaling activities. The helical bundle structures of type II IFN cytokines, such as IFN, IL-22, and IL-10, differ from those of type I four-helix cytokines in some situations, resulting in entangled dimers [122,133]. Nevertheless, the fundamental structural element of receptor CHR engaging with cytokine α-helical faces is seen in all of these various cytokine classes and continues to serve as the principal structural model for cytokine engineering investigations (Figure 5) [134].

## 5. Inflammatory Signaling Involved in Respiratory Diseases

**Figure 6:**
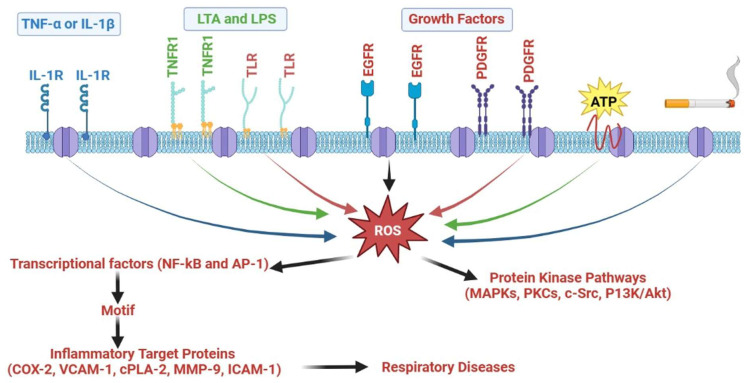
Graphical representation of the inflammatory signaling cascades in respiratory conditions. Numerous identified inflammatory targeting molecules (i.e., cPLA2, COX-2, VCAM-1, ICAM-1, and MMP-9), are linked to inflammation activation cascades triggered by diverse stimuli, including LPS, LTA, cigarette smoke, ATP, IL-1, and TNF-α. Additionally, by approaching transcription variables such as AP-1 and NF-κB in response to extracellular triggers, MAPKs, PI3K/Akt, NOX/ROS, GFTKR, PKCs, and SFKs constitute parts of signaling pathways that modulate the expression of inflammatory markers linked to respiratory conditions. Abbreviations: Akt, protein kinase B; AP-1, activator protein 1; ATP, adenosine triphosphate; COX, cyclooxygenase; EGFR, epidermal growth factor receptor; ICAM-1, intracellular adhesion molecule-1; IL, interleukin; LPS, lipopolysaccharide; LTA, lipoteichoic acid; MAPK, mitogen-activated protein kinases; MMP, matrix metalloproteinases; NOX, nicotinamide adenine dinucleotide phosphate oxidase; NF-κB, nuclear factor kappa-light-chain-enhancer of activated B cells; PDGFR, platelet-derived growth factor receptor; PKC, protein kinase C; PLA2, type II phospholipase A2; P13K, phosphatidylinositol-3-kinase; ROS, reactive oxygen species; SFK, Src family kinase; TLR, toll-like receptor; TNF, tumor necrosis factor; VCAM-1, vascular cell adhesion molecule-1. Figure created using BioRender.com (Premium Version) (accessed on 17 November 2024).

Inflammatory molecules, such as matrix metalloproteinases, COX-2, cPLA2, intracellular adhesion molecule-1 (ICAM-1), and vascular cell adhesion molecule-1 (VCAM-1), are implicated in the pathophysiology of respiratory disorders, including asthma and COPD (Figure 1, Figure 2 and Figure 3). The expression of these inflammatory molecules is also influenced by a range of inflammatory signaling cascades, including mitogen-activated protein kinases (MAPKs), protein kinase C (PKC), phosphatidylinositol-3-kinase (P13K)/Akt, Src family kinases (SFKs), ROS/Nicotinamide adenine dinucleotide phosphate oxidase (NADPH) oxidase (NOX), AP-1, epidermal growth factor receptor (EGFR), platelet-derived growth factor receptor (PDGFR), and NF-κB (Figure 6).

### 5.1. MAPKs

Growth factors, cytokines, neurotransmitters, and pharmacological and physical stimuli are significant mediators for the stimulation of MAPKs, which are pertinent aspects of the signaling network. These exogenous stimuli trigger transient reactions in the airway that alter smooth muscle activation, and they may also induce prolonged reactions that alter the integrity of the airway [135]. Transformations in the regulation of numerous genes that encode proteins that mediate cell–cell communication, cell-cycle regulation, intracellular signaling cascades, and extracellular matrix modification cause either transient or prolonged episodes in airway modification [136]. p38 MAPKs, c-Jun N-terminal kinases (JNKs), and ERKs are the three families of MAPKs that have been explored in mammals. Biochemical stressors, such as cytokines, LPS, UV radiation, and growth hormones, trigger JNK and p38 MAPK. ERK is triggered by a variety of stressors, such as cytokines and growth factors [137]. Therefore, it suppresses allergen-induced airway inflammatory responses by hindering MAPK activation via pharmaceutical or molecular strategies. Additionally, individuals with asthma revealed elevated immunostaining for phosphorylated (p)-JNK, p-ERK, and p-p38 MAPK, particularly in smooth muscle and airway epithelial cells [136]. The lateral region of the columnar epithelium is where p38 MAPK phosphorylation is most prominently exhibited. For this distinct cellular class, it is plausible that p38 MAPK regulates baseline metabolic functions. A preliminary investigation of p38 MAPK revealed that TNF-α and IL-1β cause monocytes to become activated [138]. Similarly, it has been established that restricting the p38 MAPK circuit has anti-inflammatory implications by reducing the production of TNF-α, IL-6, and IL-1β [139]. There is an intriguing possibility that IL-1β promotes prostaglandin E2 (PGE2) generation and COX-2 upregulation in airway smooth muscle cells via the p38 MAPK and ERK systems [140]. The JNK antagonist SP600125 substantially reduced the levels of chemokine ligand 5, IL-13, IL-4, and TNF-α in lung supernatants from rats with allergen-induced prolonged lung inflammation [141]. Consequently, MAPKs are essential in modulating inflammation in the lungs and airways.

### 5.2. Protein Kinase C

PKCs have significant involvement in the contraction, apoptosis, flexibility, hypertrophy, migration, multiplication, and secretion of cells in the lungs [142]. PKC, an imperative group of enzymes implicated in transmission cascades, phosphorylates substrate preferentially at threonine/serine domains with a minimum of 11 distinct isoforms. The heterogeneous modulation of PKC isoforms by diacylglycerol, phospholipids, and calcium (Ca^2+^) results in heterogeneity in their morphology, intracellular distribution, expression, precursor utilization, and stimulation mechanisms [142]. The three categories of PKC isoforms are atypical, novel, and conventional. The morphological and physiological alterations in the homologous motifs C1–C4 serve as the basis for this categorization [143]. PKCs are central regulatory gatekeepers in persistent respiratory disorders, including COPD and asthma. Inflammation of the bronchi, bronchoconstriction, and the formation of mucus have all been linked to PKCs [143]. Specifically. PKCδ modulates the generation of proinflammatory molecules via localized bronchi epithelium cells [144]. Improved PKCδ expression promotes the production of NF-κB-dependent proinflammatory mediators in human bronchial epithelial cells, whereas the presence of a dominant-negative PKC variant hinders this mechanism. In individual bronchial smooth muscle cells, PKCα, γ, μ, τ, βI, and ε are distributed in the cytoplasm, and PKC βII is expressed in the bilayer under baseline circumstances [145]. When administered to bronchial smooth muscle cells, the pro-inflammatory potent vasodilator peptide bradykinin (BK) triggers PKCα, ε, γ, and βI. Through a PKCε-dependent pathway, BK also triggers PGE2 deposition and COX-2 enzyme production in individual bronchial smooth muscle cells. COPD patients have elevated PKCα, which is suggested to contribute to the hypertrophy and multiplication of bronchial smooth muscle cells [143]. Propagating individual bronchial smooth muscle cells enhances PKCτ expression. However, PKC plays an integral role in modulating the actions of proinflammatory mediators by phosphorylating cytosolic PLA2 (cPLA2) and inducing the formation of arachidonic acid through phospholipids, which in response leads to the formation of functional eicosanoids in stimulated cells [146]. In individual bronchial interstitial fibroblasts, PKC is an important fibrosis modulator. Interstitial fibroblasts partially encode three PKCs, viz*.,* PKCα, ε, and δ [143]. Proinflammatory mediators, including IL-6, IL-β, and TNF-α, are released in response to LPS activating specific PKCs [147]. Furthermore, thrombin stimulates intracellular Ca^2+^ and triggers selective PKCs [148]. TNF-α has been shown to augment MM-9 production in A549 cells via a PKCα-dependent mechanism [149]. This evidence suggests that PKCs are important in modulating inflammatory and respiratory disorders.

### 5.3. P13K/Akt

The PI3K protein series is associated with a comprehensive range of cellular processes, including expansion, migration, multiplication, and longevity. Therefore, PI3K deregulation has been attributed to the development or advancement of a number of clinical conditions, especially those involving the respiratory system [150]. PI3Ks are classified into three types based on their architecture and lipid target selectivity [151]. Category I PI3Ks have received the most experimental emphasis. Cellular membrane receptors (e.g., G-protein-coupled receptors (GPCRs)), insulin, and growth factors trigger category I PI3Ks. Category II PI3Ks include the isotypes α, β, and γ, which are distinguished by the inclusion of a C2 motif at the C-terminal. The single target for category III PI3Ks is phosphatidylinositol. Category IA and category IB PI3Ks are subcategories of category I PI3Ks. In the context of configuration, PI3K IA occurs as heterodimeric units that couple specific regulatory subunits with enzymatic p110 subunits [151]. Ras and receptor TKs are significant downstream regulatory pathways for PI3K IA. The p110 enzymatic subunit functionalized with the p101 regulatory subunit and the downstream signaling of Ras and GPCRs constitute the PI3K IB category [151]. Current information on the pharmacological activity of PI3K in pulmonary inflammation has been substantially advanced by the two clinically available PI3K blockers, LY294002 and Wortmannin [152]. Additionally, the intratracheal administration of LY294002 significantly attenuated ovalbumin (OVA)-induced increases in eosinophil numbers, cumulative cell numbers, and chemokine ligand 11, IL-13, and IL-15 concentrations in BAL fluid and markedly prevented OVA-induced bronchial mucous secretion and tissue eosinophilia [153]. Moreover, this experiment reported that LY294002 significantly restricted OVA-induced Akt serine phosphorylation, a downstream target of PI3K. Additionally, other investigations have shown that Wortmannin and LY294002 reduce bronchial hyperresponsiveness and eosinophilic bronchial inflammation in a mouse asthmatic paradigm [153]. Thus, it has been suggested that PI3K modulation may be advantageous in alleviating the inflammation of asthmatic bronchi. However, PI3K is also activated in response to ROS generation. LY294002 was reported to attenuate chemokine-induced ROS production in phagocytic cells, and investigations utilizing PI3K mutant rodents further validated this observation [152]. As a result, the PI3K group is extensively implicated in innumerable types of pulmonary and bronchial inflammation.

### 5.4. Src Family Kinases

Molecules known as signaling SFKs have traditionally been documented to modulate important cellular functions, such as metastasis, longevity, migration, and multiplication [154]. The Src tyrosine kinase (TK) family has nine components classified as nonreceptor TKs. Src, Yrk, Yes, and Fyn are abundantly distributed, while Lck, Fgr, Lyn, Hck, and Blk are distributed in relatively constrained sequences [155]. Components of the Src TK family are triggered by physiological stressors and the activation of a number of cellular membrane receptors, including GPCRs, integrin receptors, and TK receptors [155]. Additionally, we established that IL-1β or TNF-α promotes ICAM-1 and VCAM1 activity in human bronchial smooth muscle cells via a c-Src-dependent mechanism [154]. The modulation of IL-6/PGE2/COX-2-dependent bronchial aggravation by c-Src through ROS/NOX has also been reported [156]. The bilateral blockade of these kinases with both SU6656 and PP2 (selective small-molecule Src TK inhibitors) dramatically lowered LPS-induced serum and respiratory chemokine and cytokine concentrations and mitigated LPS-dependent microvascular permeability and respiratory damage [157]. Furthermore, an emerging avenue of exploration is the significance of Src family TKs in inflammatory reactions.

### 5.5. ROS/NOX

ROS, which are byproducts of regular biological metabolism, function as secondary mediators. To sustain cells safe from oxidative exposure throughout biological circumstances, ROS help maintain cellular “redox equilibrium”. Additionally, the stimulation, longevity, multiplication, and organ activity of cells depend on the modulation of oxidative balance. Furthermore, exaggerated ROS generation, which is often induced by proinflammatory cytokine overstimulation, decreases either NOX or xanthine oxidase, and respiratory chain complexes lead to oxidative damage. Oxidative load is a destructive phenomenon that disrupts the lungs and bronchi, culminating in a variety of inflammatory respiratory injuries and disorders [154]. ROS are produced intracellularly by a variety of processes, such as those related to cytochrome P450, xanthine oxidase, the NOX complex, and mitochondrial biogenesis [158]. Being the predominant ROS-producing enzyme, NOX is a membrane-bound bifunctional enzymatic complex found in both nonphagocytic and phagocytic cells [159]. There are two primary functions for ROS generated by NOX. Initially, phagocytic cells demand superoxide radicals generated by NOX2 to undergo a respiratory surge that kills microorganisms. Considering the modulation of cellular signaling, NOX’s second function is significant [159]. ROS produced by NOX can selectively and persistently modify half-life, protein function, and distribution [154]. In experiments conducted in vitro involving bronchial and airway epithelial cells and macrophages, it has been documented that ROS stimulate the genomic production of inflammatory markers, such as TNF-α and IL-1 [160,161]. Individuals experiencing asthma exhibit an augmented production of ROS, including hydroxyl radicals, hydrogen peroxide (H_2_O_2_), and superoxide anions. Multiple bronchial types of cells, especially antigen-presenting cells (i.e., eosinophils, neutrophils, and macrophages), have revealed an augmented generation of ROS in asthma [162]. The extent of bronchial hyperreactivity, as assessed by methacholine exposure, is linked with abnormal ROS generation. Therefore, oxidative load significantly modulates inflammatory pathways in respiratory and bronchial disorders by overexpressing dimeric signaling molecules, such as NF-κB and AP-1, and consequently the expression of proinflammatory markers, such as cPLA2, ICAM-1, MMP-9, COX-2, and VCAM-1 [162].

### 5.6. Activator Protein-1

AP-1 is a dimeric transcriptional regulator normally composed of Fos (Fra-1, Fra-2, Fos-B, c-Fos) and Jun (Jun B, c-Jun, Jun D) proteins that interact with specific promoter regions. Different cytokines (TNF-α and IL-1β) and PKC can trigger AP-1 through many forms of MAPKs and TKs, which in turn initiate a pathway of intracellular kinases [87]. Specific stimuli induce the Fos gene to be expressed more efficiently, which leads to more Fos protein synthesis. Additional stimuli induce c-Jun phosphorylation via triggered kinase enzymes, hence enhancing stimulation. Current findings show that c-Jun and sirtuin 1 (SIRT1) are effectively coupled, inhibiting the transcription function of AP-1 and lowering MMP-9 levels [163]. Most significantly, it was revealed that SIRT1 hindered the transcription function of AP-1, which led to the production of PGE2 and the upregulation of COX-2, reducing c-Jun/c-Fos acetylation mediated by p300 [164]. As a result, AP-1 could be essential in modulating the transcription of different inflammatory mediators. There is an indication that the epithelial cells in asthmatic bronchi exhibit c-Fos more invariably [165]. Thus, AP-1 is highlighted as a significant contributor to respiratory afflictions.

### 5.7. Growth Factor Tyrosine Kinase Receptor

Membrane-bound TK receptors are essential for effective cellular metabolism, tissue regeneration, and maturation. Asthma progression has also been attributed to abnormalities in receptor signaling and transcription mechanisms [154]. The PDGFR and epidermal growth factor receptor (EGFR) groups of TK receptors have gained interest as plausible therapy targets for respiratory afflictions, as these receptors have been shown to perform important functions in persistent tissue repair in respiratory fibrosis, bronchitis, and asthma [154]. Multiple morphological alterations, such as mesenchymal cell hypergenesis, maturation, and extracellular matrix formation, are triggered by the EGFR pathways of basal mesenchymal cells, including smooth muscle cells, myofibroblasts, and fibroblasts, as respiratory afflictions advance [154]. The PDGFR pathway transmits signaling for cell longevity, motility, and development, and is typically expressed in mesenchymal cells. Heparin-binding EGF-like growth factor, betacellulin, heregulin, amphiregulin, TGF-α, and EGF are examples of EGFR mediators that are identified in the individual bronchial epithelium [166]. Investigations have noted the overexpression of multiple EGFR mediators in conditions such as asthma and COPD. By stimulating EGFR, *Pseudomonas aeruginosa* bacterial homogenate triggers the synthesis of mucin in individual bronchial epithelial cells (NCI-H292) [166]. Additionally, it has been reported that ROS drives PDGFRα stimulation through c-Src group kinases [149]. Information is emerging that p47^phox^ is phosphorylated by PKC in a manner necessary for the synthesis of ROS in response to PDGF, which is required for the regulation of MAPKs [154]. These investigations indicate that growth factor TK receptors may be significant for modulating the regulation of genes implicated in inflammation.

### 5.8. NF-κB

NF-κB is considered the key modulator of inflammatory conditions due to its crucial involvement in both the progression and clearance stages of inflammation [154]. Generally, the p65/RelA and p50 components of NF-κB form a heterodimeric structure. NF-κB is triggered by a variety of exogenous stressors, such as viruses, environmental particulates (PM10s), mitochondrial damage, and cytokines, including IL-1β and TNF-α [154]. In a mouse paradigm of ROS-induced transient airway damage, supplemental H_2_O_2_ also stimulated NF-κB. L-2-oxothiazolidine-4-carboxylate treatment markedly attenuated NF-κB trafficking into the nuclei and the production of cytokines, chemokines, and adhesion markers [167]. By restricting NF-κB stimulation in individual bronchial epithelial cells, a potential cyclin-dependent kinase antagonist has been indicated to attenuate the TNF-α-induced transcription of cellular adhesion markers [168]. Interestingly, researchers have also reported that heme oxygenase-1 upregulation suppresses NF-κB stimulation and oxidative load, which are sensitive to TNFR1 and prevent TNF-α-mediated bronchial aggravation [169]. These findings exemplify that NF-κB controls the synthesis of inflammatory proteins in bronchial and pulmonary inflammation and damage in a significant manner.

## 6. Current Cytokine-Targeted Therapeutic Implications

Targeted therapy is a novel therapeutic approach with considerable promise for treating severe chronic respiratory disorders, for which there are currently few effective therapeutic options [170]. Although the prognosis for many of these diseases is foreboding, targeted therapy shows promise for the subset of patients that respond to treatment and holds great potential for the future of medical care. The pathogenesis of several respiratory diseases, including asthma and COPD, has been linked to cytokine dysregulation [171]; however, less is known about the inflammatory mediators that are involved in COPD. Nevertheless, since the inflammatory process differs significantly from asthma, other cytokines and chemokines are likely involved, and therapeutic approaches need to be reformed. There is a current focus on finding novel drugs that inhibit the progression of airway limitation in COPD and establishing more specialized asthma treatments. These innovative therapeutic strategies are aimed at using cytokine and chemokine inhibitors and include glucocorticoids, tacrolimus, mycophenolate-helper lymphocyte (Th2)-selective inhibitors, humanized blocking antibodies against cytokines or their corresponding receptors, soluble receptors that bind secreted cytokines, low-molecular-weight receptor antagonists, and medications that obstruct the signal transduction pathways triggered by cytokines [172,173,174]. Alternatively, asthma and COPD may be treated with specific cytokines that decrease the allergic inflammatory process [175,176]. Due to the prevalence and severity of airway inflammation in chronic lung diseases, there is increased interest in identifying the factors that drive disease pathogenesis and development. Th2 cytokines are the primary targets for biological therapies for chronic airway diseases associated with eosinophilic inflammation. For example, as asthma is characterized by an eosinophilic inflammatory response orchestrated mainly by Th2-derived cytokines, this raises the possibility that limiting their release may be useful in producing therapeutic effects. Studies in experimental animals, such as mice with a deletion of Th2-specific cytokine genes, have supported this substantially. However, there are yet to be studies providing evidence that Th2 cytokine levels are elevated in the airways, and Th2 is unlikely to be relevant in COPD [177,178].

The eosinophilic inflammation associated with asthma is orchestrated by IL-5 [179,180]. The eosinophilic response to allergens and the resultant airway hyperresponsiveness (AHR) are significantly reduced in IL-5 gene knockout mice, confirming the efficacy of the method of suppressing IL-5. Anti-IL-5-blocking antibodies have been used to achieve this, and animal asthma models, including primates, are inhibited by these antibodies in terms of eosinophilic inflammation and AHR [181,182]. Clinical trials have been conducted since 2000 with the inhibitor mepolizumab, which was authorized for use in anti-IL-5 therapy for asthma by the Food and Drug Administration (FDA). Mepolizumab has been demonstrated to encourage a decrease in eosinophils, acute exacerbations, and the requirement for steroids [183,184]. Reslizumab and benralizumab are two additional anti-IL-5 antibodies that have recently received FDA approval for their safety and effectiveness in lowering asthma exacerbations [184,185]. The use of benralizumab and mepolizumab in phase 3 trials is being investigated as an IL-5 therapy for COPD indications. The initial findings revealed that mepolizumab treatment decreased COPD exacerbations, while benralizumab treatment had a modestly positive effect on lung function [186].

IgE production by B lymphocytes and eosinophil migration to the airways depend on IL-4 [187]. The distinct ability of IL-4 to induce Th2 differentiation places it at a pivotal and proximal point in the allergic response, making it an attractive target for inhibition. In mice, IL-4-blocking antibodies prevented allergen-induced AHR, goblet cell metaplasia, and pulmonary eosinophilia [188]. Therefore, blocking IL-4 may be useful in preventing allergy disorders, and soluble IL-4Rs are currently being tested in clinical settings. In individuals with moderately severe asthma, a single nebulized dosage of these receptors reverses the decline in lung function via inhaled corticosteroid cessation [189]. The IL-4 receptor inhibitor dupilumab has resulted in notable improvements in exacerbations and lung function in asthmatic patients who are already on dual therapy with inhaled corticosteroids and long-acting β adrenergic receptor agonists [190]. These findings are similar to the IL-5 treatment strategies for COPD. Dupilumab recently received FDA approval for its use in treating asthma; however, its effectiveness in treating COPD has not yet been evaluated.

There is mounting evidence that, irrespective of eosinophilic inflammation, IL-13 mimics many of the characteristics of asthma in mice, including AHR and mucus hypersecretion, and potently stimulates the release of eotaxin from airway epithelial cells [191,192]. IL-13 signals via the IL-4R chain, but can also activate several intracellular pathways by stimulating IL-13R1, making it a potential target for creating novel treatments. Lebrikizumab and tralokinumab, two anti-IL-13 therapies, were tested in phase 3 clinical asthma trials, but the inhibitors had little to no impact on lung function or reducing exacerbations [193].

Alternatively, proinflammatory cytokines, particularly IL-1β and TNF-α, may amplify the inflammatory response in asthma and COPD and corelate to disease severity. This suggests that blocking IL-1β or TNF-α may have beneficial effects, particularly in severe airway disease. Asthma-related airways express more IL-1, which activates several inflammatory genes [194]. There are no IL-1 inhibitors with low molecular weights, but IL-1RA, a naturally occurring cytokine, binds to IL-1R and inhibits IL-1 action [195]. In laboratory animals, IL-1RA lessens allergen-induced AHR; however, it does not appear that human recombinant IL-1RA is useful in managing asthma [196]. Studies on the function of IL1 in COPD have not yet been published. Through the activation of NF-κB, AP-1, and other transcription factors, TNF is produced in asthmatic airways and may be crucial in increasing asthmatic inflammation [197]. In asthma sufferers’ provoked sputum, TNF levels are noticeably elevated [198]. Furthermore, there is scientific evidence that COPD patients who lose weight may exhibit increased TNF release from circulating cells, which may be due to TNF inducing skeletal muscle apoptosis, resulting in the distinctive cachexia seen in certain patients with severe COPD [199]. Even in patients who are comparatively insensitive to steroids, blocking humanized monoclonal antibodies directed against TNF (infliximab) and soluble TNF receptors (etanercept) has shown impressive clinical responses in rheumatoid arthritis and inflammatory bowel disease [200,201]. Clinical trials are currently being conducted on these antibodies and soluble TNF receptors as a logical approach to treating asthma, especially in patients with severe disease (Table 3). They may also help treat severe COPD, especially in patients with malaise and cachexia. Low-molecular-weight TNF inhibitors are being investigated due to the issues with antibody-based treatments. An MMP-related TNF-converting enzyme is essential for releasing TNF from the cell surface. Oral TNF inhibitors made of low-molecular-weight TNF-converting enzyme inhibitors are currently being developed [202].

### 6.1. Future Cytokine-Targeted Therapeutics for Respiratory Diseases

Respiratory conditions and diseases have been documented to have increased levels of a variety of proinflammatory mediators, which makes them ideal treatment candidates. The following sections further explore cytokine-targeted therapeutic implications in respiratory diseases.

#### 6.1.1. Anti-IL-1β

IL-1β is a proinflammatory mediator that is upregulated in respiratory conditions (e.g., cystic fibrosis bronchiectasis, COPD, and asthma) [74]. Neutralizing IL-1β antibodies reduced the bacterial burden and pulmonary inflammation induced by *Pseudomonas aeruginosa* in cystic fibrosis rodents and enhanced IL-1β activation upon exposure to *Pseudomonas aeruginosa* [203]. In contrast to pauci-granulocytic and eosinophilic asthma, neutrophilic asthma has been reported to have elevated IL-1β levels [66]. He et al. explored the connection between the possibility of developing genomic variants and asthma in IL-1RA and IL-1-511C/T in a meta-analysis of 15 retrospective investigations [204]. The IL-1-511C/T variant was not identified; however, the IL-1RA variant was linked to an elevated incidence of asthma, which was irrespective of age or race [204]. The effectiveness of IL-β inhibitors for asthma and COPD has only been investigated in modest clinical experiments. Canakinumab is a human IgGκ monoclonal antibody with a high affinity that obliterates the biocompatibility of IL-1β. There has only been one randomized double-blind experiment in asthmatic individuals, and it used two single treatments on days 1 and 15 in moderately asthmatic individuals. Allergen exposures were undertaken on days 0 and 28, while individuals were permitted to continue consuming other anti-asthmatic therapeutics. The findings revealed that canakinumab reduced delayed asthmatic responsiveness by 28%. Canakinumab also markedly suppressed systemic IL-1 concentrations for the interval that was monitored after a single administration. Although this experiment was short and had a small sample size (16 individuals), the outcomes were favorable and optimistic [205]. The primary function of IL-1β is to trigger the inflammasome and promote resistance toward respiratory diseases; hence, precautions should be taken while employing IL-1 antagonistic therapies. Individuals with persistent inflammatory respiratory conditions may become more vulnerable to acute bronchial infection and relapses when IL-1 is blocked. As a result, it is important to extensively explore and implement specific treatments to decrease the inflammation process in definite individual categories.

#### 6.1.2. Anti-IL-6

Primary human bronchial epithelial and inflammatory cells both have the ability to release IL-6 upon exposure to a spectrum of diverse stressors [206,207]. The impairment that advances with aging, pulmonary capacity decline, and a rise in the incidence of COPD are all hallmarks of “inflammaging” [208]. Proinflammatory mediators implicated in aging, particularly IL-6, have been related to poor medical conditions and fatality, and it is thought that they cause a sustained, moderate stimulation of long-term inflammation [209]. There are other hypotheses for age-related increases in systemic IL-6 concentrations, such as enhanced oxidative damage or chronic viral infestations (i.e., cytomegalovirus and herpes), which are reported in the weak and aged [210]. Nixon et al. revealed that in cystic fibrosis bronchiectasis, IL-6 mucus concentrations were inversely correlated with forced vital capacity and forced expiratory volumes for one second, with IL-6 declining following antibiotic therapy [211]. Serum concentrations of IL-6 and BAL fluid in asthmatic individuals were shown to be higher [212]. According to these investigations, IL-6 appears to have substantial involvement in the advancement of asthma and the subsequent gradual deterioration in pulmonary function. In various inflammatory conditions (mainly Crohn’s disease and rheumatoid arthritis), inhibiting IL-6 receptors and their antibodies has been investigated. Tocilizumab, a neutralizing antibody for the IL-6 receptor, was reported in experimental studies to reduce IL-6 concentrations and ameliorate disease severity ratings and symptomatology in rheumatoid arthritis. The antibody was authorized in 2010; however, up to this point, it has not been investigated in persistent inflammatory bronchial disorders [213]. Accordingly, emphasizing IL-6 might be a noteworthy and effective therapeutic approach to reduce pulmonary capacity impairment, systemic inflammation linked to COPD, and persistent bronchial aggravation.

#### 6.1.3. Anti-IL-8

Inflammatory airway epithelial cells are a key origin of IL-8, a cytokine that is pivotal for neutrophil trafficking [214]. Improvements in fibroblast growth factor-23 and TGF-β were shown to augment airway epithelial cell IL-8 expression [215]. The upregulation of IL-8 in cystic fibrosis bronchiectasis has also been found to be induced by NF-κB, with an augmentation during the initial stages of *Pseudomonas aeruginosa* invasion [216,217]. The airway epithelial cells of individuals with cystic fibrosis were also triggered by IL-1β to release IL-8 [218]. Additionally, IL-8 has been identified as a significant factor in the pathogenesis of COPD. COPD airway epithelial cells have greater initial concentrations of IL-8, which effectively causes mucous hypersensitivity by expressing the mucosal genes MUC5B and MUC5AC [219]. TNF-α and smoking cigarettes have both been detected to effectively augment macrophage IL-8 secretion [220]. This implies that smoking cigarettes is an effective promoter for abnormal IL-8 secretion in COPD passageways [221]. Moreover, it has been established that neutrophils and systemic IL-8 increases result in the acute exacerbation of COPD, with IL-8 having detrimental implications on pulmonary functioning [222]. The outcomes of inhibiting IL-8 have been investigated in COPD, but to date no appreciable therapeutic benefits in individuals have been reported [223]. Notably, CXC chemokine receptor 2 (CXCR2) is a neutrophil chemoattractant and has been proven to support IL-8 synthesis. It is a therapeutic candidate, since research has shown that its inhibitor suppresses IL-8 secretion. Individuals with asthma administered navarinxin (a CXCR2 receptor antagonist) showed a substantial decrease in serum and mucus neutrophils without any impairment in pulmonary functioning [224]. Navarinxin exhibited marginal pulmonary function changes in experimental COPD investigations [225]. Danirixin, an additional CXCR2 antagonist, is being explored for therapeutic application [65]. While these therapeutics appear intriguing, extensive investigation and validation are still required.

#### 6.1.4. Anti-IL-4

Respiratory disease pathogenesis is regulated by IL-4, which promotes Th2 cell maturation and proliferation, the phenotypic switching of B cells to IgE production, eosinophil mobilization, mast cell growth, and mucosal fibroplasia [226]. Additionally, by augmenting the synthesis of fibronectin and collagen, IL-4 is directly implicated in the restructuring of the bronchi. Numerous studies examining the effectiveness of anti-IL-4 therapeutics in the management of asthma have produced contradictory findings [227]. The inhibition of IL-4 or its receptor in mouse paradigms of allergen-sensitized asthma has been demonstrated to reduce pulmonary swelling, blood IgE concentrations, and bronchial hyperactivity to cholinergic drugs (i.e., methacholine). It also suppresses IL-5 production from T cells and eosinophil infiltration into the bronchi [228,229]. Furthermore, although highly accepted, pascolizumab (humanized anti-IL-4 monoclonal antibody (mAb) IgG1) is ineffective in asthmatic individuals [230]. Individuals experiencing chronic, mild-to-extreme asthma and sputum or blood eosinophilia have recently been investigated with dupulimab (human mAbs that target the IL-4 receptor α subunit). In comparison to a sham group, dupilumab substantially reduced the rate of asthma complications throughout the discontinuation of inhaled β_2_ sympathomimetics and corticosteroid treatment. This was followed by a significant improvement in pulmonary capacity and a decline in the concentrations of Th2 cell-linked biomarkers (e.g., eotaxin-3), exhaled NO, and eosinophils [190].

#### 6.1.5. Anti-IL-5

IL-5 is essential for the development, differentiation, and stimulation of eosinophils [231]. As a result, pharmacological approaches that target anti-IL5 may be beneficial in therapy for eosinophilic asthma presentations [232]. Multiple preliminary investigations using asthmatic experimental animals have been performed in this direction. In addition, the administration of allergen-induced mice with the anti-IL-5 neutralizing antibody (TRFK-5) was effective in preventing eosinophil inflow through the bronchi [233]. Benralizumab, reslizumab, and mepolizumab are a few additional antibodies that have recently been discovered [234]. Mepolizumab (humanized recombinant IgG1-κ mAbs that target IL-5) has been demonstrated to be effective and efficacious in suppressing eosinophil counts in the serum and bronchi in a variety of clinical investigations conducted in diverse demographics of individuals with mild-to-severe recurrent obstructive asthma [235,236]. Furthermore, substantial refinements in asthma episodes, pulmonary capacity, and airway hyperactivity did not coincide with these outcomes.

Mepolizumab has recently been evaluated in a few subgroups of prolonged recurrent asthma, which is known for periodic relapses and bronchial eosinophilia that is resistant to systemic and/or inhaled corticosteroid treatments [237]. Collectively, the findings from these two short studies show that mepolizumab was highly sustained throughout the course of a 12-month therapy interval and significantly reduced asthmatic acute episodes and eosinophil concentrations in serum and produced mucus [238]. An intriguing humanized mAb (IgG4-κ), reslizumab, is a potential anti-IL-5 biosimilar agent in parallel to mepolizumab. Reslizumab has recently been demonstrated to dramatically enhance pulmonary capacity and reduce sputum eosinophils in individuals with eosinophilic asthma compared to sham treatment, producing a favorable shift toward superior asthmatic treatment. The greatest concentrations of blood and sputum eosinophils, which were linked to the existence of chronic rhinosinusitis, were prevalent in a subset of individuals, and these individuals were characterized by the strongest anti-asthmatic responses to reslizumab [239]. As a result, these studies highlight the significance of precise phenotypic screening in an effort to customize anti-asthmatic therapy to the unique biosimilar and symptomatic factors of various disease presentations.

#### 6.1.6. Anti-IL-9

In respiratory diseases, IL-9 is upregulated and is responsible for promoting an increase in goblet cell numbers and mast cell proliferation [240]. Hyperactivity and inflammation of the bronchi were attenuated in mice following IL-9 suppression [241]. Additionally, MEDI-528 (humanized mAb that targets IL-9) demonstrated a satisfactory tolerability record in controlled phase 2a studies conducted in patients with moderate asthma and simultaneously elicited a signal favoring recovery in terms of asthma severity ratings and clinical recurrence patterns. The second of these experimental investigations demonstrated that 50 mg of MEDI-528, given subcutaneously biweekly, can exhibit a preventive response toward exercise-mediated bronchospasm [242].

#### 6.1.7. Anti-IL-13

Since IL-13 is involved in numerous attributes of bronchial swelling and restructuring, such as the mobilization of basophils and eosinophils, the expansion of bronchial smooth muscle cells and pulmonary fibroblasts, IgE production, and extra mucus secretion, it has become a pivotal target for the advancement of novel anti-asthmatic targeted therapies. In preclinical experimental models of long-term allergic asthma, anti-IL-13 therapy significantly reduced bronchial morphological abnormalities, bronchial eosinophilia, and IgE production [243]. Anrukinzumab (humanized mAb anti-IL-13) effectively suppressed delayed asthmatic episodes triggered by allergens in clinical studies within 14 days, but not 35 days [244]. Lebrikizumab (humanized mAb inhibiting IL-13) has also been demonstrated to have a beneficial anti-asthmatic effect that is defined by the upregulation of IL-13-inducible genes, including periostin (matricellular protein) [245]. Lebrikizumab was effective in improving respiratory capacity in individuals with moderate-to-severe asthma who had elevated periostin serum concentrations. The percentage improvements in forced expiratory volume for one second recorded for 12 weeks were 5.5% overall in the lebrikizumab treatment group, 8.2% in the elevated-periostin group, and 1.6% in the low-periostin group, compared to reference data. This indicates potential readily observable biomarkers that may be regularly utilized in clinical practice to pinpoint unique asthmatic subtypes, marked by a prominent destructive involvement of IL-13 [246]. Tralokinumab (humanized mAb blocking IL-13), which is presently undergoing clinical investigation, has improved pharmacokinetic parameters and a satisfactory tolerability potential [247].

#### 6.1.8. Anti-IL-17 and Anti-IL-23

In airway biopsies retrieved from individuals exhibiting chronic asthma, IL-17F and IL-17A (two proinflammatory mediators vitally implicated in neutrophilic activation and bronchial restructuring) are markedly elevated [248]. From this perspective, it has become interesting that anti-IL17 mAbs reduce the number of lymphocytes, eosinophils, and neutrophils found in BAL fluid in mouse models of allergen-induced asthma [249]. Currently, clinical phase II investigations are determining the effectiveness and stability of secukinumab (humanized mAb against IL-17A) and brodalumab (humanized mAb working specifically against the IL-17 receptor) in individuals with chronic asthma that is not satisfactorily managed by inhaled β_2_-sympathomimetics and corticosteroids [250]. The application of antibodies against the mediator IL-23, which regulates the production of IL-17, may be an alternative and promising therapeutic treatment strategy. By suppressing this mediator, lymphocyte, eosinophil, and neutrophil migration into hypersensitive mouse bronchi was markedly reduced [251]. Risankizumab (humanized mAb blocking IL-23) is still undergoing clinical studies for the treatment of chronic asthma [65]. IL-17 is also implicated in immunological resistance toward pathogens and carcinogenesis. Therefore, inactivating this mediator may raise the incidence of secondary infestations, and the occurrence of cancer and experimental approaches must be carefully examined [252].

#### 6.1.9. Anti-TSLP, Anti-IL-25, and Anti-IL-33

Respiratory conditions present elevated numbers of thymic stromal lymphopoietin (TSLP), IL-33, and IL-25, which are primarily expressed by the bronchial epithelium and are essential for triggering Th2-dependent inflammatory events [253]. Currently, it is hypothesized that these mediators may be promising candidates for innovative anti-asthmatic therapeutics. The attenuation of Th2-mediated sensitive airway swelling in mice by an anti-IL-25 monoclonal antibody has been documented [252]. Additionally, antibodies targeting the human IL-33 receptor have the potential to decrease the mouse allergen-induced inflammatory response and bronchial hyperactivity [254]. In adult airway epithelial cells, these antibodies may also significantly reduce the production of IL-17F [255]. A significant reduction in murine allergen-induced bronchial inflammation has also been documented as a result of antibody-mediated TSLP protein inactivation [256].

#### 6.1.10. Anti-IL-27

IL-27 is a heterodimeric cytokine expressed by macrophages and monocytes that has been implicated in the etiopathogenesis of chronic corticosteroid-induced asthma. Similarly, IL-27 values in the bronchi of individuals with chronic neutrophilic asthma are augmented. Furthermore, glucocorticoid receptor nuclear trafficking, a crucial intracellular process for the pharmacological and biological effects of corticosteroids, was hindered by IL-27 in mouse pulmonary macrophages. IL-27 may be a promising candidate for novel treatment approaches designed to improve the management of chronic, corticosteroid-resistant asthma [256].

#### 6.1.11. Anti-GM-CSF

Respiratory diseases upregulate the monomeric glycoprotein GM-CSF, which is essential for eosinophil viability and maturation [257]. An inhalant dose of a goat anti-mouse GM-CSF polyclonal antibody effectively reduced bronchial swelling, extra mucus secretion, and bronchoconstriction in a mouse paradigm of allergen-induced asthma [258]. Subsequently, namilumab (human IgG1 mAb blocking GS-CSF) was designed, and it has demonstrated its ability to rapidly reduce the survivability and stimulation of local adult eosinophils [259].

### 6.2. Safety of Anti-Cytokine Therapies

Randomized controlled trials have been used to evaluate adverse outcomes connected to drugs. Patients who received omalizumab and those who received a placebo saw similar rates of treatment-related adverse events. Even in a cohort of patients with chronic oral corticosteroid usage, mepolizumab was not linked to a higher incidence of adverse events when compared to a placebo [260]. Rare instances of hypersensitive reactions may occur. Anaphylactic responses following injections of reslizumab were uncommon and well tolerated [261]. Nasopharyngitis was the most frequent side effect seen with benralizumab, but its incidence was not appreciably different from that of the placebo group [185]. Injection-site reactions and upper respiratory tract infections were the most frequent side effects of dupilumab when compared to the placebo. There have been reports of persistent occurrences of symptomatic hypereosinophilia; this has been seen in 4–25% of individuals treated with dupilumab [262]. One explanation for this hypereosinophilia is that blocking the IL-4/IL-13 pathway inhibits the eotaxin-3, vascular cell adhesion protein 1, thymus, and activation-regulated chemokines without also blocking eosinophilopoiesis in the bone marrow. This decreases eosinophil migration and causes an increase in blood eosinophils. Therefore, it appears that the existence of hypereosinophilia (>1500 μL^−1^) is advantageous when selecting the best biologic. Blood eosinophilia should be regularly checked in patients with severe asthma when dupilumab is started. Clinical and immunological responses to tocilizumab as an adjuvant treatment for severe pediatric persistent asthma were demonstrated in a recent case report with few side effects [263]. There was no discernible difference between the tezepelumab and placebo groups in terms of the types and rates of adverse events. The inclusion of patients with severe asthma in registries will provide clinicians with information on long-term safety, and the longer-term follow-up of patients treated with biologics is crucial to verify the lack of subsequent occurrences.

### 6.3. Ongoing and Completed Clinical Trials for the Management of Respiratory Diseases Based on Cytokine Therapy

The data for ongoing and completed clinical trials investigating the management of various respiratory diseases based on cytokine therapy are presented in Table 4 with the disease conditions and interventions.

### 6.4. Future Therapeutic Options and Challenges

The notion of remission has been created recently, and several definitions have been applied. Remission is obviously defined as the absence of exacerbations, stopping systemic corticosteroid treatment, and controlling asthma symptoms [264]. The inclusion of the fourth criterion, improvement in lung function, is still open to controversy because certain patients with chronic illnesses and fixed airflow obstructions may not be able to normalize or improve their lung function while they are asymptomatic and free from exacerbations. Developing an agreement on the definition of remission also requires a consideration of the patient’s perspective. Astegolimab, an anti-ST2 medication, has been the subject of current research [265]. In individuals with low blood eosinophil levels, this medication, which targets the IL-33 pathway, resulted in a marginally better reduction in exacerbation; this suggests that the IL-33 pathway may be more significant in type 2 low severe asthma. Itepekimab is an anti-IL-33 human IgG4P monoclonal antibody [266]. In one study, every 2 weeks for 12 weeks, 296 patients with moderate-to-severe asthma were randomly randomized to receive 300 mg subcutaneous itepekimab, itepekimab plus dupilumab (both at 300 mg), dupilumab (300 mg), or a placebo. Itepekimab and dupilumab together did not work better than either medication by itself. All four study groups had comparable rates of adverse events. In a phase 2a, multicenter, randomized, double-blind, placebo-controlled, 24-week trial, anti-IL-23 risankizumab was studied in moderate-to-severe asthma [267]. The findings of the study revealed that risankizumab exerted a biologic effect on airway immunity, which may have contributed to the poor clinical outcome. By stimulating the local release of neutrophil-mobilizing molecules, such as CXC chemokines, IL-17 plays a crucial part in the pulmonary host defense. There is debate about recent claims of IL-17’s involvement in the onset of COPD. Similarly to the evidence on COPD, Th17 cells and the cytokines they produce (IL-17A and IL-17F) seem to have a role in the pathophysiology of severe and steroid-resistant asthma [268]. When it comes to chronic inflammatory airway illnesses, IL-17 regulation represents an appealing prospective therapeutic target. The efficacy of IL-17 and its receptor antagonist in treating COPD and asthma has been studied in small clinical trials. Regretfully, patients with either condition have not shown any improvements in lung function or disease symptoms. One of the major worries is whether mAbs are cost-effective for treating critically ill patients, especially in low-income nations. Another concern is whether health insurance companies will agree to pay for the treatment. According to a retrospective analysis, it normally takes seven to eight years to develop mAbs and an additional year to receive FDA approval; a priority review might shorten the approval process by around eight months [268].

## 7. Conclusions

The respiratory tract illustrates a distinctive interface with the external environment, and the induction of effective but rigidly regulated innate immune responses is pivotal to protect against pathologies and control and eliminate infections caused by invading pathogens, while simultaneously reducing the disturbance in the complex pulmonary anatomy. Proinflammatory cytokines produced by macrophages strengthen the host’s defense against pathological conditions. Although pro-inflammatory cytokines might be favorable in controlling pathologies, their extreme rise in level can cause large-scale tissue damage. There has been viable realization regarding the contribution of similar cytokines in the expression of lung pathology among chronic pulmonary diseases.

Cytokines, including IL-4, IL-13, TGF-b, IL-17, and TSLP, assist in many chronic disease states. Increasing evidence has revealed the involvement of inflammatory proteins, including cPLA2, COX-2, VCAM-1, ICAM-1, and MMP-9, in the production and development of respiratory diseases. Additionally, these inflammatory proteins are regulated by various inflammatory signaling pathways, including PKCs, NOX/ROS, EGFR, PDGFR, c-Src, PI3K/Akt, MAPKs, AP-1, and NF-κB. However, multiple questions remain unanswered, although the general role of cytokines in respiratory diseases has been elucidated. There have been few studies investigating the role of transcription factors, such as the transcription factor NF-kB. In asthma, there is an increased expression of NF-κB in the airway epithelium, as well as alveolar macrophage expression and the generation of IL-8. Oxidative stress may also lead to the activation of NF-κB. Surprisingly, the expression of NF-κB is not increased in COPD, suggesting that NF-κB may not be needed for the transcription of cytokines. There may be important differences between asthma and COPD at the level of the transcriptional control of inflammatory cytokines [269]. Further studies are mandatory for the direct comparison of various pulmonary diseases and to concentrate on patients who show clinical features of any two respiratory disorders. Whether various respiratory disease conditions have common mechanisms or similar predisposing factors can be ascertained by the further investigation of cytokine expression. Additionally, this may reveal some common pathways in respiratory conditions. The functions of cytokines at the time of localized infection and systemic infection may have crucial differences. Several findings have stressed the emphasis of clinically relevant animal models to fully understand the role of cytokines in the pathogenesis of lung bacterial infection. The cytokine network can be locally modulated to serve as a significant addition to antibiotic therapy. The translation of exceedingly assuring experiments in animals into clinical trials fails to keep pace. It is important to note that the management of proinflammatory cytokines in respiratory diseases should be tailored to the specific disease and individual patient, taking into consideration factors such as the underlying cause of the disease, the severity of the symptoms, and the patient’s overall health. One complication is that cytokines are functionally pleiotropic and redundant, and as a result, targeting an individual cytokine may have a less than expected effect or have undesired side effects. Another hardship is that animal models do not entirely mimic human disease. In addition, the exploration of the role of inflammatory proteins in widespread respiratory diseases is essential to identify possible therapeutic targets. Advancement in designing novel inhibitors with high specificity and no major adverse effects is essential. The option of targeted delivery of inflammatory protein inhibitors directly to the airway and lungs should also be explored, as it may have fewer side effects.

Thorough studies are needed to fully elucidate the role of inflammatory signaling molecules in pulmonary diseases to develop highly specific inhibitors. Until now, blocking specific cytokines or their receptors has been disappointing in clinical studies, which in turn suggests that a broader spectrum of anti-inflammatory effects is needed to treat individuals with severe respiratory diseases.

## Figures and Tables

**Table 1:** Classification of cytokines, their origin of secretion, and roles during inflammation.

Cytokines	Key Secreting Cells	Roles During Inflammation	References
IL-1	Endothelial cells, epithelial cells, macrophages, monocytes, dendritic ells	Induces inflammation, fever, and acute phase response, and accelerates production of neutrophils	[25,41,60]
IL-2	CD4^+^ and CD8^+^ T cells	Activates NK cells and cytotoxic T cells and increases production of other cytokines	[25,41,60]
IL-3	CD4^+^ T cells	Produces growth factor for hematopoietic stem cells	[41,60]
IL-4	CD4^+^ Th2 cells and mast cells	Promotes differentiation of Th2 cells and aids in growth and survival of mast cells, B cells, and T cells	[41,60]
IL-5	CD4^+^ Th2 cells	Stimulates growth and progression of eosinophil	[25,41]
IL-6	Endothelial cells, macrophages and T lymphocytes	Induces the liver to create acute-phase inflammatory response mediators	[41,60]
IL-7	Stromal cells of bone marrow	Plays a role in adaptive immunity by promoting the growth and division of thymocytes and pre-B cells	[41,60]
IL-8 (CXCL8)	Endothelial cells, macrophages, neutrophils	Plays a role in adaptive immunity, controls lymphocyte migration and neutrophil infiltration, and attracts neutrophils and T lymphocytes	[41,60]
IL-10	Macrophages, Th cells, regulatory T cells, dendritic cells	Reduces inflammation by preventing Th1 cells and the release of IL-12 from activated macrophages and dendritic cells	[41,60]
IL-12	Dendritic cells and macrophages	Increases the cytotoxicity of NK cells in innate immunity and triggers the development of Th1 cells in adaptive immunity	[41,60]
Type-I IFN (IFN-α, IFN-β)	Fibroblasts, macrophages, dendritic cells, endothelial cells, epithelial cells	NK cell activation, viral replication inhibition, and increases MHC-I molecule expression on virus-infected cells	[41,44,60,61,62]
IFN-γ	CD4^+^ and CD8^+^ T lymphocytes and NK cells	Promotes the production of MHC-I and II and the processing and presentation of antigens; activates macrophages in innate immune responses and adaptive cell-mediated immune responses	[41,44,61,62]
TNF-α	Macrophages and T cells	Brings about inflammation, causes acute phase reaction and fever, and induces neutrophil and endothelial cell activation and cellular apoptosis	[43,60]
TGF-β	T cells	Induces cytotoxicity and phagocytosis	[43,60]
Chemokines	Endothelial cells, macrophages and T cells	Leukocyte migration from the blood and tissues is controlled and stimulated by chemokines	[41,53,56]
G-CSF	Endothelial cells, fibroblast and macrophages	Encourages the production of growth factors and neutrophil maturation in inflammatory reactions	[41,52,60]
M-CSF	Macrophages and T cells	Encourages mononuclear phagocyte maturation and growth factors	[41,52,60]
GM-CSF	Endothelial cells, fibroblast, macrophages, and T cells	Activates mature granulocytes and encourages the maturation and expansion of neutrophil, eosinophil, and monocyte cells	[41,52,60]

Abbreviations: G-CSF, granulocyte colony-stimulating factor; GM-CSF, granulocyte monocyte colony-stimulating factor; IL, interleukin; IFN, interferon; M-CSF, monocyte colony-stimulating factor; MHC, major histocompatibility complex; NK, natural killer; Th, T-helper; TNF: tumor necrosis factor.

**Table 2:** Major lung diseases and the corresponding cytokines that are involved in their pathogenesis and development.

**Lung Disease**	**Cytokines Involved**	**References**
Asthma	CCR2-5 agonist, CXCR2-3 agonist, EGF, eotaxin, GM-CSF, IL-1 β, IL-4, IL-5, IL-6, IL-8, IL-9, IL-13, IL-17, IL-25, IL-33, NGF, RANTES, SCF, TGF-β, TNF-α, TSLP, VEGF	[63,65,66,67]
COPD	CCR2 agonist, CCR3 agonist, CCR5 agonist, CXCR2 agonist, CXCR3 agonist, EGF, GM-CSF, IL-1β, IL-4, IL-6, IL-8, IL-12, IL-17, IL-18, IL-32, IFN-γ, TGF-β, TNF-α, TSLP	[63,65,68,69,70,71]
COVID-19-associated lung disease	IL-1β, IL-8, IL-6, IL-17, TNF-α	[72,73]
Cystic fibrosis bronchiectasis	IL-1β	[74,75,76,77,78]
Lung cancer	IFN-γ, IL-1β, IL-6, IL-8, IL-10, IL-17, IL-18, IL-22,TGF-β, TNF-α, VEGF	[79,80]
Pneumonia	IFN-γ, IL-lβ, IL-6, IL-8, IL-10, IL-33, MCP-1, TNF	[81,82,83,84]
Pulmonary fibrosis	CCL17, CCL18/PARC, CTGF, CXCL12/SDF-1, GM-CSF, IL-1, IL-4, IL-10, IL-13, IL-17, MCP-1, oncostatin M, PDGF, TGF- β	[63,65,72,85]
Pulmonary tuberculosis	TNF-α, IL-1β, IL-12, IL-18, IL- 23, IL-27, IL-10, IL-6, IL-17, IL-22, IFN- β, IFN- γ, TGF- β	[86]

Abbreviations: CTGF, connective tissue growth factor; EGF, epidermal growth factor; GM-CSF, granulocyte–macrophage colony-stimulating factor; IFN, interferon; IL, interleukin; MCP-1, monocyte chemotactic protein-1; PARC, pulmonary and activation-regulated chemokine; PDGF, platelet-derived growth factor; RANTES, regulated and normal T-cell expressed and secreted; SDF-1, stromal cell-derived factor-1; TGF, transforming growth factor; TNF, tumor necrosis factor; TSLP, thymic stromal lymphopoietin; VEGF: vascular endothelial growth factor.

**Table 3:** Description of available cytokine-targeted therapeutics for treating respiratory diseases that are in clinical trials (data were retrieved from ClinicalTrials.gov using search terms such as cytokine and respiratory disease) (accessed on 10 November 2024).

Targeted Cytokine	Intervention Molecule	Type of Antibody	Disease Condition	Clinical Efficacy	Status	Company/Organization	Clinical Trials Associated
IL-5	Mepolizumab	Humanized, IgG1 monoclonal antibody	COPD, high levels of sputum eosinophils, and severe eosinophilic asthma	Reduces moderate and severe exacerbations	Phase III	GlaxoSmithKline	NCT02105961 NCT02555371 NCT01691859 NCT02135692
			Severe bilateral chronic rhinosinusitis	Improved nasal polyp size and nasal obstruction	Phase III	GlaxoSmithKline	NCT03085797
			Severe asthma and comorbid conditions	Controls asthma, improved lung function and health-related quality of life, as well as various comorbid pathologies	Phase II	GlaxoSmithKline	NCT01000506
					Phase III	GlaxoSmithKline	NCT01691521
					Phase III	GlaxoSmithKline	NCT01842607
					Phase III	GlaxoSmithKline	NCT02281318
			Hypereosinophilic syndrome	Decreased disease flares and blood eosinophil count	Phase III	GlaxoSmithKline	NCT03306043 NCT02836496
	Benralizumab	Humanized, afucosylated anti-IL-5 receptor α monoclonal antibody	Severe eosinophilic asthma, COPD	Reduced the exacerbations in severe COPD	Phase III	AstraZeneca	NCT01914757
					Phase III	AstraZeneca	NCT01928771
					Phase II	MedImmune LLC	NCT01227278
					Phase III	AstraZeneca	NCT02155660
					Phase III	AstraZeneca	NCT02138916
			Chronic rhinosinusitis	Reduced NP score, nasal blockage, and difficulty with sense of smell	Phase III	AstraZeneca	NCT03401229
			Severe eosinophilic asthma	Improved nasal polyposis and lung function	Phase III	AstraZeneca	NCT03170271
			Severe, uncontrolled asthma	Found to be safe and effective in long-term studies	Phase III	AstraZeneca	NCT02258542
			Severe asthma	Clinical remission is achievable and reduced exacerbation rates	Phase III	AstraZeneca	NCT02075255
	Reslizumab	Humanized monoclonal antibody	Eosinophilic granulomatosis with polyangiitis (EGPA)	Found to be safe and effective in EGPA	Phase II	National Jewish Health	NCT02947945
			Late-onset eosinophilic asthma	Reduction in asthma exacerbations and improved lung function	Phase III	Teva Branded Pharmaceutical Products R&D, Inc.	NCT01287039 NCT01285323
IL-4	Dupilumab	Human anti-interleukin-4 receptor α monoclonal antibody	Uncontrolled Asthma	Lower rates of severe asthma exacerbation, better lung function	Phase III	Sanofi	NCT02414854
			Glucocorticoid-dependent severe asthma	Decreased the rate of severe exacerbations and increased FEV1	Phase III	Sanofi	NCT02528214
			Chronic rhinosinusitis with nasal polyps	Improved upper and lower airway outcome measures	Phase III	Sanofi	NCT02912468 NCT02898454
			Persistent asthma	Improved lung function and decreased Th2-associated inflammatory markers	Phase II	Sanofi	NCT01312961
IL-13	Lebrikizumab	Anti-interleukin-13 monoclonal antibody	Uncontrolled asthma in adolescent patients	Reduced asthma exacerbation rates	Phase III	Hoffmann-La-Roche	NCT01875003
			Idiopathic pulmonary fibrosis	Benefits lung function	Phase II	Hoffmann-La-Roche	NCT01872689
			Adults with asthma	Improved lung function with high levels of serum periostin	Phase II	Genentech, Inc.	NCT00930163
	Tralokinumab	Anti-interleukin-13 monoclonal antibody	Moderate-to-severe asthma	Improved lung function with no improvement in ACQ-6	Phase IIa	MedImmune LLC	NCT00873860
TNF-α	Infliximab	Anti-TNF-α antibody	Asthma	Results are not posted	Phase II	Imperial College London	NCT00278083

Abbreviations: IL, interleukin; COPD, chronic obstructive pulmonary disease; EGPA, eosinophilic granulomatosis with polyangiitis; FEV1, forced expiratory volume; ACQ-6, asthma control questionnaire score 6.

**Table 4:** Clinical trials based on cytokine therapy for the management of respiratory diseases (data were retrieved from ClinicalTrials.gov using search terms such as cytokine and respiratory disease) (accessed on 10 November 2024).

NCT Number	Title	Conditions	Interventions	Phase	Status	Company/Organization
NCT00001908	T-Cell Cytokine Changes During IL-4 Receptor Treatment for Asthma	Asthma Hypersensitivity	--	--	Completed	National Institute of Allergy and Infectious Diseases
NCT00455767	Safety and Efficacy Study of Depelestat in ARDS Patients	Fibrosis Lung Disease Respiratory Disorders Acute Respiratory Distress Syndrome (ARDS) Pulmonary Fibrosis Inflammation	EPI-hNE4, Placebo	Phase II	Completed	Debiopharm International SA
NCT00753103	Anti-Cytokine Therapy for Vasculitis	Wegener’s Granulomatosis Renal Limited Vasculitis Microscopic Polyangiitis	Infliximab, Cyclophosphamide, Prednisolone, Azathioprine, Mycophenolate Mofetil, Methylprednisolone	Phase II	Completed	University Hospital Birmingham NHS Foundation Trust
NCT01253941	Effects of Mud Bath Therapy in Chronic Obstructive Pulmonary Disease	COPD	Mud Bath Therapy	N/A	Completed	Fondazione Salvatore Maugeri
NCT02113072	Recurrent Wheezing in Infants: Risk Factors and Prevention With Probiotics	Respiratory Tract Diseases, Wheezing	Beclomethasone, Probiotics, Placebo	Phase III	Completed	Universidade Federal de Pernambuco
NCT02557958	Chronic Obstructive Pulmonary Disease Transcription Factor and Cytokine Study	COPD	Azithromycin, Placebo	Early Phase I	Completed	NYU Langone Health
NCT03595488	Dupilumab for Aspirin-exacerbated respiratory disease	Aspirin-Exacerbated Respiratory disease	Dupilumab	Phase II	Completed	Rochester General Hospital
NCT04102813	Deep Diaphragmatic Breathing: Neurobiological and Anti-inflammatory Effects	Unrecognized Condition	Functional Therapy	N/A	Completed	Azienda Ospedaliera Universitaria Policlinico Paolo Giaccone Palermo
NCT04374149	Therapeutic Plasma Exchange Alone or in Combination with Ruxolitinib in COVID-19-Associated CRS	Cytokine Release Syndrome COVID-19	Therapeutic Plasma Exchange Ruxolitinib	Phase II	Completed	Prisma Health Upstate
NCT04445272	Clinical Trial to Evaluate the Effectiveness and Safety of Tocilizumab for Treating Patients With COVID-19 Pneumonia	COVID-19	Tocilizumab	Phase II	Completed	Fundacion SEIMC-GESIDA
NCT04457349	Therapeutic Plasma Exchange in Resistant Cytokine Storm of COVID-19	COVID-19	Therapeutic Plasma Exchange	N/A	Completed	Alexandria University
NCT04485169	Therapeutic Plasma Exchange for Coronavirus Disease-2019 Triggered Cytokine Release Storm	COVID-19 Cytokine Release Syndrome	Therapeutic Plasma Exchange	N/A	Completed	UNICEF
NCT05164692	Effects of Nasal-spraying LiveSpo Navax in Treatment of Acute Respiratory Infections in Children	Acute Respiratory Tract Infections	LiveSpo Navax, 0.9% NaCl Physiological Saline	N/A	Completed	National Children’s Hospital, Vietnam
NCT05378022	Effects of Nasal-spraying LiveSpo Navax in Treatment of Influenza Virus in Children	Acute Respiratory Tract Infections	Combination Product: LiveSpo Navax, 0.9% NaCl Physiological Saline	N/A	Completed	National Children’s Hospital, Vietnam
NCT05562843	Autologous Cellular Therapy With PRP-PC in Chronic Lung Diseases: An Observational Study LI-004	COPD ILD	Autologous Cellular Therapy with PRP-PC	N/A	Completed	H-CYTE

Abbreviations: ARDS, acute respiratory distress syndrome; COPD, chronic obstructive pulmonary disease; ILD, interstitial lung disease; COVID-19, coronavirus 2019; IL, interleukin; N/A, not applicable.

## Data Availability

Not applicable.
